# Nutritional benefits of sourdoughs: A systematic review

**DOI:** 10.1016/j.advnut.2022.10.003

**Published:** 2022-12-16

**Authors:** Léa Ribet, Robin Dessalles, Corinne Lesens, Nele Brusselaers, Mickaël Durand-Dubief

**Affiliations:** 1 Baking Science, Lesaffre Institute of Science & Technology, Lesaffre, Marcq-en-Barœul, France; 2 Robin Dessalles EI, Lyon, France; 3 Global Health Institute, Antwerp University, Antwerp, Belgium; 4 Centre for Translational Microbiome Research (CTMR), Department of Microbiology, Tumor and Cell Biology, Karolinska Institute, Karolinska Hospital, Stockholm, Sweden; 5 Department of Head and Skin, Ghent University, Ghent, Belgium; 6 Discovery & Front End Innovation, Lesaffre Institute of Science & Technology, Lesaffre, Marcq-en-Barœul, France

**Keywords:** bread, clinical trials, fermentation, glycemic response, lactic bacteria, microbiota, satiety, sourdough, yeast

## Abstract

Food fermentation using sourdough—i.e., consortia of lactic bacteria and yeasts—is increasingly considered among the public as a natural transformation yielding nutritional benefits; however, it is unclear whether its alleged properties are validated by science. The aim of this study was to systematically review the clinical evidence related to the effect of sourdough bread on health. Bibliographic searches were performed in 2 different databases (The Lens and PubMed) up to February 2022. Eligible studies were randomized controlled trials involving adults, healthy or not, given any type of sourdough bread compared with those given any type of yeast bread. A total of 573 articles were retrieved and investigated, of which 25 clinical trials met the inclusion criteria. The 25 clinical trials included a total of 542 individuals. The main outcomes investigated in the retrieved studies were glucose response (*N* = 15), appetite (*N* = 3), gastrointestinal markers (*N* = 5), and cardiovascular markers (*N* = 2). Overall, it is currently difficult to establish a clear consensus with regards to the beneficial effects of sourdough per se on health when compared with other types of bread because a variety of factors, such as the microbial composition of sourdough, fermentation parameters, cereals, and flour types potentially influence the nutritional properties of bread. Nonetheless, in studies using specific strains and fermentation conditions, significant improvements were observed in parameters related to glycemic response, satiety, or gastrointestinal comfort after bread ingestion. The reviewed data suggest that sourdough has great potential to produce a variety of functional foods; however, its complex and dynamic ecosystem requires further standardization to conclude its clinical health benefits.

**Statements of significance**

Scientific studies have already highlighted that sourdough fermentation can improve nutrient bioaccessibility or reduce the GI of bread; however, it remains unclear whether the effect of sourdough fermentation on cereals translates to beneficial effects in a clinical setting. Our systematic review reveals the difficulty in establishing a clear consensus with regards to the beneficial effects of sourdough per se on health and suggests that sourdough has great potential to produce a variety of functional foods; however, its complex and dynamic ecosystem requires further standardization to conclude its clinical health benefits.

## Introduction

Fermentation is used as a means of natural transformation for enhancing food properties such as preservation or palatability, i.e., flavor or texture [1]. It involves the controlled growth of microorganisms in conditions allowing enzymatic conversions of specific food components [2,3]. Among the different types of food fermentation, the sourdough process, using a combination of lactic acid bacteria (LAB) and yeast, has been traditionally used as a leavening agent in baking. Initially, sourdough was prepared by letting the microorganisms naturally present in the raw food or the direct environment develop within a mixture of flour and water, generally at the ambient temperature over several hours. A sample of this fermented dough is then reinoculated in a new mixture of flour and water, and the process is repeated; this method is referred to as “backslopping” [4]. Public awareness regarding nutrition and health as well as the renewed interest in simple traditional food processes, such as sourdough bread, has increased over the past decades. Along with this increased popularity, sourdough bread fermentation is claimed to promote various health benefits, such as better digestibility and enhanced nutritional content. Although scientific studies have already highlighted that sourdough fermentation can improve nutrient bioaccessibility and reduce the GI of bread [1,4,5], it remains unclear whether the effect of sourdough fermentation on cereals translates to beneficial effects in a clinical setting and, if so, whether these clinical benefits apply in a real-life situation given that study design and comparator choice often limit extrapolation of results. Therefore, the objective of this work was to systematically review the clinical evidence related to the benefits of sourdough-fermented bread on health.

## Methods

Bibliographic searches were performed with no time restriction on PubMed and The Lens databases [6] (https://www.lens.org/) using the following keywords in a number of combinations: “sourdough AND bread AND (human OR subjects OR volunteers)” (see Supplementary Table 1). In this work, 2 researchers (RD, MD-D) independently evaluated the quality of the included studies. Articles published up to February 2022 and written in the English language were screened. The Population, Intervention, Comparator, Outcomes, and Study Design method was used to define the selection criteria. Briefly, clinical studies were eligible if they included healthy or unhealthy adult subjects (aged >18 y) (Population); used any form of bread and baked goods fermented with sourdough as intervention (Intervention) compared with any form of bread fermented with yeast only (Comparator); evaluated the effect of the intervention on any clinical health outcome (Outcomes); and were randomized controlled or nonrandomized experimental clinical studies published in peer-reviewed journals (Study Design). The PRISMA methodology was applied [7].

The risk of bias in each study was evaluated using a number of components known to be potential sources of bias in interventional studies [8,9]. Studies were classified as being at low, medium, or high risk of bias on the basis of authors’ judgment of the potential bias arising from each individual component. Study characteristics and quality assessment results are detailed in Supplementary Figs. 1 and 2 and Supplementary Table 2. Although the taxonomy of the genus *Lactobacillus* has recently evolved [10], we decided to maintain the former nomenclature for clarity purposes because most of the studies reviewed in this work used this form.

## Results

The PRISMA flow diagram is reported in Supplementary Fig. 3. The searches yielded 573 articles, of which 25 clinical trials met the inclusion criteria, published between 1995 and 2022. Glycemic control was investigated as a primary outcome in 15 studies. The remaining studies focused on appetite (*N* = 3), gastrointestinal markers (*N* = 5), and cardiovascular markers (*N* = 2). Most studies were from the European Region (*N* = 18), mostly from Italy (*N* = 8), Finland (*N* = 4), and Sweden (*N* = 3), with the 3 remaining studies being conducted in Denmark, Croatia, and the United Kingdom. The other works were from Canadian (*N* = 5), Israeli (*N* = 1), and New Zealander (*N* = 1) research teams. In terms of the risk of bias, 16 studies were considered low risk, 5 studies were considered medium risk, and 4 studies were considered high risk (Supplementary Table 2 and Supplementary Fig. 2). Studies’ sample sizes ranged from 8 to 87 subjects, representing a total of 542 individuals.

### Postprandial glucose response

Among the 20 studies assessing the effect of sourdough bread on glucose response, either as a primary (*N* = 15) or secondary outcome (*N* = 5), 14 focused on healthy individuals, 2 focused on individuals with obesity or who were overweight, 2 focused on both healthy and hyperglycemic individuals, 1 focused on subjects with impaired glucose tolerance, and 1 focused on individuals with type 2 diabetes. In total, measures of glucose response were available for 369 individuals (Supplementary Table 2). Studies usually reported results as AUC or incremental AUC (iAUC) (to account for the variations in baseline values) for outcomes such as glucose and insulin response.

Among 8 studies mentioning starter composition, most reported the use of *Lactobacillus* strains *Lactobacillus plantarum* (*N* = 6) and *Lactobacillus brevis* (*N* = 4). Other mentioned strains were (1 study each): *Lactobacillus* *acidophilus*, *Lactobacillus* *casei*, *Lactobacillus fermentum*, *Lactobacillus* *rossiae*, and *Lactobacillus sanfranciscensis*. According to the different studies, *Saccharomyces cerevisiae* was the most commonly used yeast as a starter, except for 1 study mentioning *Saccharomyces* *exiguous*. The grains used for bread making were mostly wheat or rye; barley, oat, or corn flours were rarely used. Twelve studies allowed the comparison of a similar bread recipe, only varying in the presence of sourdough rather than the presence of yeast fermentation, whereas the remaining 8 studies compared different recipes, such as whole-grain sourdough bread with white wheat yeast bread. In addition, 6 studies focused on commercial breads, with little or no information available on the bread-making process.

#### Healthy subjects

A total of 14 studies had measures of glycemic response available for 263 healthy subjects. In an early Swedish randomized controlled study, the effects of whole-meal yeast bread on the glucose response of healthy subjects were compared to those of the same bread with added sourdough containing *Lactobacillus* *plantarum* A1. Bread was given as part of a macronutrient- and energy-matched breakfast. The iAUC related to the glucose response was significantly lower over the whole time period (0–120 min) in the sourdough group than in the control group [11]. In 2 other studies focusing on healthy subjects, glucose response was significantly decreased over the whole assessment period (lasting from 120 to 300 min, depending on studies) in the sourdough group compared with that in the yeast group [12,13]. However, no significant differences in glucose response over the assessment period were observed in 5 other trials [[14], [15], [16], [17], [18]].

In 7 studies, the effect of sourdough bread based on whole-grain wheat or rye flour on glucose response was compared with that of baker’s yeast white wheat bread in healthy subjects. A significant reduction in glucose response over the assessment period was shown in 2 studies [12,19], whereas no differences between the 2 bread types could be identified in 5 other trials [14,18,[20], [21], [22]].

#### Subjects with obesity or metabolic diseases

Among the 6 studies, including a total of 78 subjects with impaired glucose metabolism, in an Italian study, a significant reduction in glucose AUC was observed in the first 60 min after sourdough bread intake compared with that with its yeast counterpart but not thereafter [23]. A Canadian study highlighted a significantly lower glucose iAUC (180 min) after the intake of sourdough whole-grain wheat bread than after the intake of refined wheat bread in hyperglycemic subjects but not in normoglycemic subjects [24]. Two other Canadian studies found no evidence of a difference in glycemic parameters between whole-grain sourdough bread and white wheat bread whether in normoglycemic or hyperglycemic individuals [25] or individuals with type 2 diabetes [26]. In another Canadian trial including subjects with obesity and those who were overweight, the observed glucose iAUC with sourdough white wheat bread was significantly lower than that observed with white wheat or whole wheat yeast bread [27]. Using commercial bread, the same research team found that sourdough white wheat bread was not different from baker’s yeast white wheat bread regarding its effect on glucose response in subjects who are overweight when breads were matched for delivering 50 g of carbohydrates. However, when breads were matched for mass, the glucose iAUC of the sourdough bread was significantly higher than that of the white wheat bread [28].

### Effect of sourdough on appetite and satiety

The 7 retrieved studies that focused on appetite markers as a primary or secondary outcome included a total of 147 healthy subjects (Supplementary Table 2). Five studies mentioned the bacteria present in the sourdough. The strains reported were *Lactobacillus* *plantarum* (3 studies), *Lactobacillus* *brevis* (2 studies), *Lactobacillus* *acidophilus* (1 study), *Lactobacillus* *casei* (1 study), *Lactobacillus* *sanfranciscensis* (1 study), *Lactobacillus* *rossiae* (1 study), *Streptococcus* sp. (1 study), and *Leuconostoc* sp. (1 study). The flours used for these studies were wheat or rye. One study also used a mixture of barley and wheat or organic einkorn flour. The markers of satiety in these studies were usually scores calculated on the basis of visual analog scales (VAS) for different components related to satiety (“fullness,” “hunger,” and “desire to eat”), hormonal response (ghrelin AUC), or EI at a subsequent meal. Most studies expressed the VAS results as AUC.

In 5 studies comparing sourdough breads with their yeast counterparts, no significant differences in any of the satiety parameters assessed were observed [11,15,18,19,29]. In an Italian study, sourdough bread ingestion induced a significantly higher appetite AUC and a significantly lower satiety AUC than those induced by baker’s yeast bread [13]. In another trial, a significantly lower AUC for hunger and higher AUC for satiety were found between 45 and 240 min after ingestion of a sourdough croissant in healthy subjects than those found after ingestion of its yeast counterpart [30].

Among 3 studies comparing different bread recipes with that of white wheat bread, Italian researchers observed a significantly reduced ghrelin AUC and a significantly higher satiety AUC after consumption of sourdough organic einkorn bread compared with those after consumption of white wheat bread [19]. In a Swedish trial, sourdough-fermented whole-grain (19 %) rye crispbread was found to generate lower hunger and desire to eat than those generated by refined wheat crispbread [18].

Another Swedish trial compared the effects of breads containing different combinations of sourdough (9%, 30%, or 51%) with rye (35%, 42%, or 45%) on appetite ratings in healthy subjects. It was highlighted that breads with low rye content did not induce a significant difference in hunger, fullness, or desire-to-eat ratings compared with those induced by white wheat bread, regardless of the sourdough content. However, breads with medium or high rye content did exert significant effects, regardless of the sourdough levels. The different rye breads tested did not differ significantly from one another with regards to their effects on appetite ratings [29].

### Effect of sourdough on gastrointestinal health

A total of 7 studies focused on gastrointestinal health parameters; among which, 4 focused on healthy subjects (*n* = 90) and 3 investigated the effects of sourdough breads with a reduced content of fermentable oligosaccharides, disaccharides, monosaccharides and polyols (FODMAP) or reduced gluten content, in individuals suffering with irritable bowel syndrome (IBS) (*n* = 170) (Supplementary Table 2). In the 4 studies in which information was available, the microorganisms reported to be used in sourdough were mostly *Lactobacillus* *sanfranciscensis* (2 studies), *Lactobacillus* *brevis* (2 studies), *Lactobacillus* *alimentarius* (1 study), *Lactobacillus* *hilgardii* (1 study) and *Lactobacillus* *plantarum* (2 studies). Other studies reported (1 study each) the following microorganisms: *Streptococcus* sp., *Leuconostoc* sp., and *Lactobacillus* *rossiae*. Of note, depending on the study context, the strains were selected on the basis of their known characteristics (e.g., fructan degradation).

#### Gastrointestinal comfort in healthy subjects

Among the 4 studies including healthy subjects (*n* = 90), it was reported that providing sourdough rather than baker’s yeast croissants did not affect gastric emptying but lowered both expired hydrogen AUC at 45–240 min and subjective gastrointestinal discomfort during the 0–240-min period in the sourdough group compared with in the control group [30]. In another study, sourdough bread, either made spontaneously or with a starter, significantly lowered the gastric emptying rate compared with that with baker’s yeast bread in healthy subjects. No effects of bread on gastrointestinal symptoms were reported [13]. In another study, when comparing rye sourdough bread to white wheat bread, no significant differences were reported in the gastric emptying rate in healthy subjects [20]. In a Finnish trial, the investigators reported a significantly increased frequency of slight to moderate flatulence in subjects given sourdough whole-grain rye bread or white wheat bread enriched with rye bran compared with that in subjects given white wheat bread alone; however, no significant differences in bloating, rumbling of the stomach, abdominal pain, or heartburn were reported between interventions [21].

#### Effects of low-FODMAP or low-gluten sourdough bread in IBS

A total of 3 studies included 170 individuals with IBS. A Finish research team investigated whether sourdough fermentation using specific *Lactobacillus* strains could be used to decrease FODMAP content in bread and, thus, reduce abdominal discomfort in patients with IBS. They did not report significant differences in IBS severity score (SS) or IBS quality of life values between traditional sourdough (1.1 g/100 g fructans) and low-FODMAP (0.3 g/100 g fructans) sourdough rye breads in 87 subjects with IBS. However, abdominal symptom scores and hydrogen breath concentrations significantly improved in the low-FODMAP bread group [31]. In another study, the same research team did not highlight significant differences in gastrointestinal symptoms between subjects who were given a low-FODMAP (0.06 g/100 g fructans) refined sourdough wheat bread and those who were given a refined yeast wheat bread (0.23 g/100 g fructans). However, the score related to non-GI symptoms (such as tiredness, joint symptoms, and decreased alertness) was significantly higher in the low-FODMAP sourdough group than in the yeast group [32].

A randomized controlled trial reportedly used sourdough fermentation with specific *Lactobacillus* strains in combination with fungal proteases to reduce gluten content (50% reduction of immune reactive gluten) in wheat bread and assessed whether the resulting product would reduce gastrointestinal symptoms in patients with IBS, compared with normal-gluten bread. The authors reported a significant decrease in gastrointestinal symptoms measured through VAS in the gluten-reduced sourdough bread compared with in the yeast bread with normal gluten content but no changes in IBS-SS or IBS quality of life scores [33].

### Effect of sourdough on cardiovascular outcomes

A total of 7 studies investigated the effects of sourdough bread on cardiovascular health parameters as a primary or secondary outcome (Supplementary Table 2). Four studies focused on healthy subjects (*n* = 73), and 3 studies focused on individuals with metabolic impairments (*n* = 42).

In a Canadian trial, the investigators examined the influence of whole-grain sourdough bread consumption compared with that of white wheat bread consumption on PAI-1, a biomarker of CVD that may be altered by dietary carbohydrates. After a 6-wk consumption period, no significant differences could be observed between the groups, neither in normoglycemic individuals nor in hyperglycemic individuals [24].

In an Italian study, no differences in blood lipids or inflammatory parameters were reported between the sourdough and yeast bread groups; however, LDL cholesterol was significantly decreased compared with baseline in both groups by 10.6% and 8.53%, respectively [34]. Accordingly, no impact of sourdough bread compared with that of white wheat bread on blood lipids was observed in other studies involving healthy subjects [15,19], individuals with impaired glucose tolerance [23], or subjects who were overweight [26]. However, in another randomized controlled trial including healthy subjects, a significant decrease in both total and LDL cholesterol levels was found after consumption of whole wheat sourdough bread compared with that after consumption of white wheat yeast bread [22].

## Discussion

**FIGURE 1:**
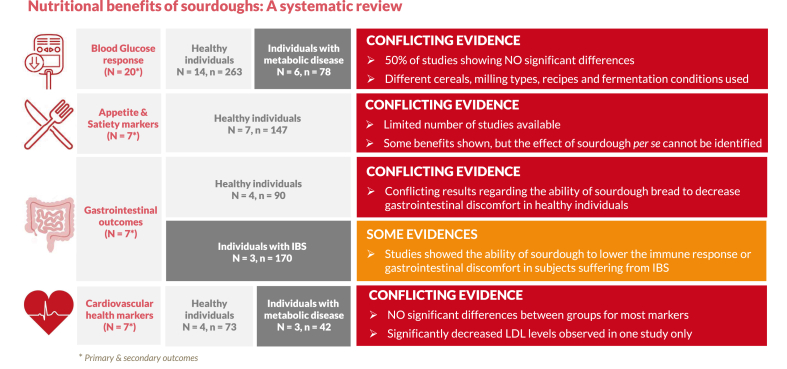
Summary of the reviewed clinical trials included in this study according to the health outcomes and target population.

The objective of the present paper was to systematically review the clinical evidence investigating the effects of sourdough bread consumption on various health measurements relative to other sources of bread. The review included 25 interventional clinical trials, mostly on healthy subjects, using commercial sourdough breads or laboratory-made sourdough, made either spontaneously or with different combinations of LAB and yeast as a starter. The main health parameters assessed were glycemic response, satiety, and gastrointestinal and cardiovascular health. In weighing the evidence, it is currently not possible to conclude that using sourdough instead of baker’s yeast for fermentation during bread making would be sufficient to highlight significant benefits on health in a clinical setting. More than 50% of the studies comparing sourdough bread with white wheat bread did not find significant differences in the glycemic response of healthy individuals between the groups. Several studies showed evidence of a significant effect of sourdough bread on some appetite ratings compared with control bread; however, the effect of sourdough could not be separated from that of grain and flour type in these studies. Regarding gastrointestinal outcomes, sourdough fermentation was shown to reduce FODMAP content in bread, making it more acceptable for patients with IBS, although the strains mentioned were selected specifically for this purpose (see the summary of the results, Figure 1).

This heterogeneity in the results of these studies is likely because of the diversity of sourdough preparations across studies, which may have yielded products with different nutritional characteristics and metabolic effects. Indeed, sourdough is a complex and dynamic ecosystem. Although the most commonly occurring microorganisms in sourdough made according to the traditional backslopping method are the association of the LAB *Lactobacillus* *sanfranciscensis* and the yeast *Candida humilis*, the spontaneous development of other species has extensively been reported. These notably include *Lactobacillus* *fermentum*, *Lactobacillus* *plantarum,* and *Lactobacillus* *brevis* for LAB or *Candida*, *Kazachstania*, *Rhodotorula*, and *Saccharomyces* genera for yeast [2,4,5,35]. The presence of 1 type of microorganism over another also depends on fermentation conditions, notably time and temperature [36]. The starters used for industrial or research purposes also vary widely and most commonly contain *Lactobacillus* *plantarum*, *Lactobacillus* *brevis*, and *Lactobacillus* *sanfranciscensis*; however, the use of other genera, such as *Bifidobacterium*, *Enterococcus*, or *Leuconostoc*, has also been reported [5]. In the present work, when mentioned, the strains reportedly used for sourdough preparation were mostly *Lactobacillus* *plantarum* and *Lactobacillus* *brevis* but also included *Lactobacillus* *acidophilus*, *Lactobacillus* *casei*, *Lactobacillus* *sanfranciscensis*, or *Lactobacillus* *fermentum*, whereas the yeast strain in sourdough was mostly *Saccharomyces* *cerevisiae*. Unfortunately, information was not available for nearly 50% of the studies. Hence, noting the diversity of the sourdough ecosystem and the wide variety of industrial practices, the attribution of a nutritional or health effect to sourdough per se is currently not possible. Establishing this relationship is especially complex because studies also differed in fermentation, baking conditions and in the cereal and flour types used.

Most studies investigated whether sourdough bread could impact postprandial glucose response in adult subjects [37]. However, based on the studies reviewed in this article, whether sourdough fermentation per se would have a significant impact on postprandial blood glucose response is equivocal. This might be explained by the differences in design, measurement methods, and sample size and also by the presence of a variety of confounding factors, modifications to which during the bread-making process are known to influence the glycemic response to bread.

Early studies have indeed suggested that the effect of sourdough on blood glucose response was mediated by the production of organic acids during the fermentation process, which would decrease gastric emptying rate and, therefore, delay glucose response [11,38]. More recently, Darzi et al. [15] did not report an effect of propionate added to sourdough bread on postprandial glycemic response. In addition, the impact of sourdough bread on gastric emptying seemed inconsistent in other studies [13,20,30]. Variations in the starch content and structure are known to influence glucose and insulin responses [39]. Sourdough fermentation was reported to decrease the rapidly digestible starch content in bread [40], and this starch fraction was positively correlated to the estimated GI of sourdough bread, whereas the opposite was found for slowly digestible and resistant starch contents [41]. Interestingly, sourdough fermentation with a starter (*Lactobacillus* *brevis*, *Lactobacillus* *plantarum*, and baker’s yeast) was found to yield a bread with a higher resistant starch content than that with spontaneous sourdough fermentation; however, an impact of fermentation type on the estimated GI of bread was apparent only for whole-grain wheat bread, and not for white wheat bread [42]. Starch structure and organization also seem to play a role: increased gelatinization and swelling of starch granules may occur as a result of sourdough fermentation, making starch more available for digestion. This supposedly results in an increased GI [43,44]. Conversely, it was found that sourdough fermentation of rye products results in the formation of an amylose layer, which has been suggested to inhibit starch hydrolysis, thus potentially decreasing postprandial glucose response [45]. Proofing, baking time and temperature, and storage have also been reported to affect starch structure and, potentially, the glycemic response to bread [38,41,46,47]. Different patterns of starch degradation during sourdough fermentation may also release several types of fermentation metabolites, notably dextrins, maltotriose, maltose, and glucose, whose amount in the final bread could impact its GI in different directions [44].

Independent of sourdough fermentation, the inclusion of fiber or fiber-rich whole-grain or whole-meal flour in the manufacturing of breads is known to influence the postprandial glucose response [48]. In this respect, the choice of flour is a major determinant of the effect. The flour type and final bread density were reported to be larger contributors to the GI of bread than the rising method or leavening agent [43,49]. Similarly, the effect of sourdough bread on appetite ratings seems to be mediated by the fiber content of the flour [29]. It is worth noting that the nutritional content of the endosperm fraction of wheat grain is not homogeneous; it varies according to a gradient from the inner to the outer parts, the latter having, for instance, a higher amylose to amylopectin ratio. This indicates that grain processing and milling could also affect the nutritional content of the grain and may also act as a confounder in the relationship between sourdough bread and health [50]. Among the other determinants of the glycemic response to bread, it was reported that the microbiota characteristics of each individual could predict the glycemic response to bread [22].

Because of all these potential determinants, it is difficult to establish that the sourdough process would be predictive of a beneficial effect on glycemic control. It is well known that replacing high-GI diets with low-GI diets is useful for both the management of metabolic disease in unhealthy individuals and the prevention of disease progression in at-risk individuals [[51], [52], [53], [54], [55]]. Accordingly, there is evidence that decreased postprandial glycemia has a role in the prevention of diabetes. However, what constitutes a healthy postprandial glycemic response profile remains a subject of debate [37]. In this context, even in a case in which sourdough bread would improve postprandial glycemic response compared with white wheat bread (which is known to have a high GI), it is uncertain whether sourdough bread would be an acceptable alternative resulting in health improvement because this is also influenced, as mentioned previously, by other components in bread as well as by other components in the diet. Sourdough fermentation with specific strains in combination with other components, such as proteases, was shown to significantly reduce gluten in bread in vitro [[56], [57], [58], [59], [60]]. Such a combination has also been shown to reduce immunoreactivity in clinical trials [61,62]; however, more studies are needed to establish the interest and safety of the resulting products in patients with celiac disease. The use of this combination for bread production has also been shown to improve some gastrointestinal outcomes in patients with IBS [33]. Similarly, in vitro trials showed the ability of sourdough fermentation to significantly reduce the FODMAP content in bread [3,63], although fermentation with baker’s yeast alone was also reported to be very effective in this respect [64,65]. However, clinical evidence supporting the safety of the resulting low-FODMAP bread in patients with IBS remains to be confirmed [31,32].

Despite the lack of consistency in the scientific evidence related to the clinical benefits of food fermented with sourdough on health, a significant body of evidence demonstrated that sourdough fermentation improves micronutrient bioaccessibility, notably for minerals such as iron, calcium, magnesium, or zinc, through the reduction of phytic acid content induced by the phytate-degrading enzymes present in yeast and LAB isolated from sourdough [[66], [67], [68], [69], [70]]. The effect is especially significant for whole-grain or rye flours, which are a rich source of minerals but present high levels of phytic acid [71,72].

The fact that sourdough fermentation improves the accessibility of minerals and that higher whole-grain consumption is known to be associated with a reduced incidence of several chronic diseases [73] makes whole-grain sourdough-fermented bread a nutrient-dense food with potential functional properties on health.

This systematic review has a number of limitations. Because of the exploratory nature of this review and the methodological diversity of the included studies, it was considered inappropriate to perform a meta-analysis. As mentioned previously, the main issue relates to the heterogeneity of studies, which widely differ in terms of products assessed and design and often lack information related to products’ characterization. This heterogeneity appeared to be reflected in the results because it was not possible to establish a clear consensus regarding the effect of sourdough on the different health parameters assessed. Most of the studies investigated the effects of sourdough bread acutely, thus giving no indication of whether the potential beneficial effects observed would be sustained over time. Still, this work provides a valuable overview of the clinical studies related to the effect of sourdough bread on health and identifies a number of clinical parameters on which sourdough products made under proper standardized conditions could be beneficial, such as glycemic response or gastrointestinal immune response.

## Conclusions

The benefits of sourdough-fermented bread on glycemic response, satiety, or gastrointestinal distress were reported in some studies but not in others, and the lack of sourdough (LAB and yeasts) characterization and the high diversity of bacterial strains, fermentation conditions, or bread recipes used from one study to another make it difficult to identify the main determinants of the effects. Consequently, in the current state of the scientific literature, it remains uncertain whether sourdough fermentation per se could exert clinically significant benefits on health. Additional studies, standardized in terms of design and participants, with better characterization of sourdough-fermented bread and other health outcomes are required to determine the health benefits.

In the meantime, sourdough fermentation remains a valuable natural transformation for enhancing the texture, flavor, and stability of foods. It is known to enhance mineral accessibility of cereal products, especially those with high fiber content, thus being a tool of choice to produce high-fiber, nutrient-dense breads.

## Funding

Supported in part by Lesaffre International, Marcq-en-Barœul, France. The sponsor had no role in the study design, data analysis, or interpretation of results.

## Disclosures

NB, no conflicts of interest. RD, has temporary consulting activities for Lesaffre. LR, CL, and MD-D are employees of Lesaffre International, Marcq-en-Barœul, France.
