# Supplementary material for: Nutritional benefits of sourdoughs: A systematic review
Source: Adv Nutr. 2022 Dec 16;14(1):22–9. doi: 10.1016/j.advnut.2022.10.003 (PMC10103004; doi:10.1016/j.advnut.2022.10.003)
Supplement: Multimedia component 1 [file mmc1.pdf]

Supplementary Table 1: Detailed search strategy …… p. 2

Supplementary Table 2: Study characteristics of all 25 studies included in the systematic review - per topic ……………………………………… p. 3

- **Glucose and insulin responses** ……… p. 3 - 11
- **Appetite markers ..**…… p. 12 - 15
- **Gastrointestinal markers** ……… p. 16 - 21
- **Cardiovascular markers** …… p. 22 - 25

Supplementary Table 1: Detailed search strategy

Database	Search string	Details	Date of most recent search	Number of hits
PubMed	sourdough AND bread AND (human OR subjects OR volunteers)		October 2020 (Update February 2022)	*170*
Lens.org	sourdough AND bread AND (human OR subjects OR volunteers)	Search filtered with "journal articles" only	October 2020 (Update February 2022)	*557*

Supplementary Table 2 : Study characteristics of all 25 studies included in the systematic review - per topic

Characteristics of clinical trials investigating the effects of sourdough bread on **glucose and insulin responses**, either as primary or secondary outcome.

Author/ Year/ Reference	Location	Design	Subjects	Interventions	Strains	Fermentation conditions	Doses in starter (CFU/g flour)	Results	Risk of bias
**Liljeberg et al. 1995 (11)**	Sweden	RCT, Acute	11 **healthy** subjects<br>6 females, 5 males<br>Age: 26-48 y	Whole-meal bread (WMB: Barley/Wheat 80:20, with yeast) alone or with added:<br>▪ Sourdough<br>▪ Lactic acid (64 g)<br>▪ Ca-lactate (95 g)<br>▪ Na-propionate (62 g)<br>▪ Na-propionate, high [] (185 g)	*L. plantarum* A1 (ClasLönner AB, Lund, Sweden)<br>**Authentication**: NM	Proofed 50 min + 20 min, 38°C, 75% humidity	5.00E+07 CFU/g flour	▪ iAUC 0-45 min significantly ($p < 0.05$) lower in the sourdough *vs.* whole-meal bread alone (44.9 ± 4.3 *vs.* 57.4 ± 4.3 mmol/L x min)<br>▪ iAUC 0-120 min lower in the sourdough *vs.* whole-meal bread alone (78.6 ± 15.1 *vs.* 103.0 ± 13.6 mmol/L x min, p value not mentioned).	M
**Maioli et al. 2008 (23)**	Italy	RCT, CO, Acute	16 subjects with **impaired glucose tolerance (IGT)**<br>9 males, 7 females<br>Age: 52-75 y	▪ **Test product:** Wheat / cornflour (70:30) sourdough bread<br>▪ **Control product(s):** Wheat / cornflour (70:30) leavened with yeast	*S. cerevisiae, L. brevis SB3, L. plantarum SB24*, isolated from natural sourdough<br>**Authentication**: PCR test reported in a cited study	Proofed for 8 h, 30°C, 90% humidity	NM	▪ 30 minutes after the ingestion of sourdough bread blood glucose levels resulted significantly lower (-25%, $p = 0.048$) in comparison to those obtained with the reference bread.<br>▪ Blood glucose AUCs were significantly lower at intervals of D 0–30 min ($p = 0.02$) and D 0–60 min ($p = 0.018$) while the difference was no longer significant thereafter.	M
**Darzi et al. 2012 (15)**	United Kingdom	RCT, SB, CO, Acute	20 **healthy** subjects<br>9 males, 11 females<br>Age: 25.1 ± 4.6 y	White wheat-bread sandwich administered in breakfast:<br>▪ Propionate-rich sourdough bread (2.3 mg per g propionic acid and 2.98 mg per g calcium propionate)<br>▪ Control bread	"Domani starter culture"<br>**Authentication**: NM	NM	NM	▪ Postprandial glycaemic response was not significantly influenced by treatment nor was there a treatment x time effect (p not mentioned).<br>▪ No significant difference between treatments for postprandial insulin sensitivity (p not mentioned).	H
**Rizzello et al. 2019 (13)**	Italy	RCT	36 **healthy** subjects<br>50% females<br>Age: 25 ± 1.1 y	**Three types of refined wheat bread:**<br>▪ Traditional sourdough bread (t-SB) (i.e. without baker's yeast)<br>▪ Sourdough bread (SB) (LAB + baker's yeast)	*Lb. plantarum CR1, Lb. rossiae CR5* and *S. cerevisiae E10*<br>**Authentication**: Culture	4 h at 30 °C (step I)<br>1.5 h at 30 °C (step II)	5.10^{+7}(lactic acid bacteria)<br>5.10^{+7}(yeast)	▪ The peak following BYB ingestion corresponded to 14.3 ± 0.39 mg/mL which was ca. 5% lower ($p < 0.05$) than those reported for SB and t-SB.<br>▪ Compared to BYB, SB and t-SB generated a lower glycaemic curve ($p < 0.05$).<br>▪ The AUC of BYB was 14947 ± 416 mg × 120 min, while the areas for SB and t-SB were 11 and 25% lower ($p < 0.05$), respectively.	L

Author/ Year/ Reference	Location	Design	Subjects	Interventions	Strains	Fermentation conditions	Doses in starter (CFU/g flour)	Results	Risk of bias
				▪ Baker's yeast bread (BYB)	Collection of the Department of Soil, Plant and Food Sciences (University of Bari, Italy)				
**Pagliai et al. 2020 (34)**	Italy	RCT DB CO 4 weeks	17 **healthy** subjects<br><br>10 males, 7 females<br><br>Age: 34.6 ± 9.1 y	▪ Group A: bread made with ancient grain "Verna" and sourdough (SD)<br><br>▪ Group B: control bread, made with ancient grain "Verna" and baker's yeast (BY)	**Sourdough:** *Lactobacillus sanfranciscensis, Lactobacillus plantarum, S. cerevisiae*<br>**Control:** *Saccharomyces cerevisiae*<br><br>**Authentication**: genotypic identification (ARDRA)	NM	$2.64.10^{+8}$ (LAB)<br><br>$5.75\ 10^{+8}$(yeast)	▪ A statistically significant increase in blood glucose (+6%; p = .012) was observed after BY, while no significant variations were observed after SD.	L
**Scazzina et al. 2009 (12)**	Italy	RCT, Acute	8 **healthy** subjects<br><br>4 females, 4 males<br><br>Age: 24 ± 1 y	**4 experimental breads:**<br>▪ Whole wheat sourdough<br>▪ White wheat sourdough<br>▪ Whole wheat yeast<br>▪ White wheat yeast	**Sourdough:** NM<br><br>**Control:** baker's yeast (*S. cerevisiae*)<br><br>**Authentication**: NM	NM	NM	▪ Both the response curves and the $iAUC_{0\text{-}120\ min}$ values of the sourdough products are lower than the corresponding samples leavened with baking yeast.<br><br>▪ The statistical analysis shows that the leavening technique significantly affects glucose response when measured as IAUC ($p < 0.001$), whereas fiber content does not ($p = 0.325$).	H
**Novotni et al. 2012 (17)**	Croatia	Non-randomized, controlled study, Acute	11 **healthy** subjects<br><br>7 females, 4 males<br><br>Age: 21 to 27 y	**Gluten-free breads:** combination of rice flour (913 g), extruded corn flour (439 g), corn starch (439 g), potato starch (84 g), buckwheat flour (100 g)<br><br>**Experimental groups:**<br>▪ Control bread without sourdough<br>▪ 4 sourdough breads with the same formulation as control, with 7.5, 15,	*Lb. Fermentum*: Commercial starter PL3 (10.4 g) containing *Lb. fermentum* (Piemonte,Kirchzarten, Germany)	Fermentation cabinet at 30 °C and 95% relative humidity (RH) until reaching pH 4 ± 0.05.	$1.6 \times 10^{+7}$	**Glucose response:**<br>▪ All breads produced a peak response of glucose at 45 min after consumption (Fig. 2).<br>▪ The mean blood glucose was the highest after consumption of control and bread with 7.5% of sourdough but did not significantly differ among breads ($P > 0.1$).<br>▪ The glucose response in first 60 min was the lowest for breads with 15% of sourdough (Fig. 2), having peak significantly lower than the control ($P = 0.05$).	H

Author/ Year/ Reference	Location	Design	Subjects	Interventions	Strains	Fermentation conditions	Doses in starter (CFU/g flour)	Results	Risk of bias
				22.5 or 30 g sourdough/100 g	**Authentication**: NM			▪ Also, mean blood glucose increases during 120 min after consumption of breads with 15% and 22.5% of sourdough was lower than in other test breads (p=0.07) (Table 4).<br>**Glycemic Index:**<br>▪ Sourdough decreased bread's GI by 9, 16, 14 or 7 units, depending on its amount (7.5; 15; 22.5 or 30%, respectively). The difference in GI between all breads was significant (P = 0.09).<br>▪ Breads with 15% and 22.5% of added sourdough had low GI and were significantly lower than control bread (P = 0.07).	
**Bo et al. 2017 (19)**	Italy	RCT, DB, CO, Acute	16 **healthy** 6 males, 10 females<br><br>Age: 32.3 ± 7.4 y	**Test product(s):** Sourdough bread made with commercial wheat flour, organic wheat flour or organic einkorn flour<br>**Control product(s):** Bread made with commercial wheat flour, no sourdough	**Sourdough:** *L. acidophilus* (strain NM) *L. casei* (strain NM) *S. cerevisiae* (strain NM) *S. exiguous* (strain NM)<br><br>**Control:** NM<br><br>**Authentication**: NM	4-5 h at 28°C and 40% of relative humidity	50 g of starter culture (9.5.10^{+5} colony forming units/g flour)	▪ Glucose $AUCs_{0\text{-}120\ min}$ were significantly lower after the consumption of sourdough breads made by organic flour (p=0.01) and einkorn flour (p=0.002), compared to commercial wheat bread (Table 4).<br>▪ Glucose $AUCs_{0\text{-}120\ min}$ of sourdough wheat bread was not significantly different from that of commercial wheat bread (p=0.11) (Table 4).<br>▪ Statistically significant differences in glucose values occurred at 120 min with trade flour bread (p=0.04), at 90 and 120 min with organic flour bread (p=0.001 and p=0.02 respectively), and at 30, 60, 90, 120 min with einkorn bread (p=0.007, p=0.03, p=0.002 and p=0.01 respectively).	L
**Zamaratskaia et al. 2017 (18)**	Sweden	RCT, SB, CO, Acute	24 **healthy** subjects<br><br>13 males, 11 females<br><br>Age: 30 ± 11 y	▪ Sourdough-fermented whole grain (19 %) rye crispbread<br>▪ Unfermented whole grain rye crispbread, and<br>▪ Refined wheat crispbread (control)	**Sourdough:** « in-house culture mixture » (spontaneous)<br><br>**Control:** yeast<br><br>**Authentication**: NM	Two-step fermentation: first for 120 min at 29°C, followed by 35 min with an increase from 30 to 38°C.	NM	**Glucose response:**<br>▪ AUC (0–125 min) and AUC (0–230 min) values for postprandial glucose were similar after intake of all crispbreads (P=0.76 and P=0.06, respectively; Fig. 3, right).<br>▪ No significant difference in postprandial glucose response between the different crispbreads was found when modelling the curves (P=0.15; Fig. 3, left).<br>**Blood hormones:**<br>▪ GLP-1 AUC (0–125 min) and AUC (0–230 min) values were similar after intake of all crispbreads (P= 0.45 and P=0.29, respectively; Fig. 3, right).<br>▪ Overall effect of diet showed no significant effect on postprandial plasma total GLP-1 concentrations (P=0.09; Fig. 3, left).	L

Author/ Year/ Reference	Location	Design	Subjects	Interventions	Strains	Fermentation conditions	Doses in starter (CFU/g flour)	Results	Risk of bias
**Bondia-Pons et al. 2011 (20)**	Finland	Non-randomized study, Acute	16 **healthy**<br>13 females, 3 males<br>Age: 23 ± 3.7 y	**Test product:** Rye sourdough bread (refined) (RB)<br>**Control product:** White wheat bread (WB)	Not known (Commercial breads)<br><br>**Authentication:** NM	Not known (Commercial breads)	Not known (Commercial breads)	▪ The maximum increase in glucose responses and the AUC-glucose did not differ significantly among the breads (p values not mentioned)<br>▪ However, plasma glucose response was significantly higher at 90 min after eating of RB than WB (P = 0.045).<br>▪ Glucose concentration decreased below baseline fasting concentrations at 90 min after eating WB (P = 0.005).	H
**Tucker et al, 2010 (25)**	Canada	RCT, CO, 6 weeks	**Normoglycemic group:**<br>10 males, 4<br>Age: 53.1 ± 1.61 y<br>BMI: 26.5 ± 0.78 kg/m²<br>HOMA-IR 0.85 (0.73, 0.98)<br><br>**Hyperglycemic group:** 10 males, 4 females<br>Age: 57.4 ± 1.97 y<br>BMI: 35.7 ± 1.51 kg/m²<br>HOMA-IR 3.00 (2.60, 3.46)	**Test product:** Whole grain wheat flour (37% dw), non-wheat grains (18% dw) (commercially available), sourdough<br><br>**Control product(s):** White wheat bread (commercially available)	Not known (Commercial breads)<br><br>**Authentication:** NM	Not known (Commercial breads)	Not known (Commercial breads)	Glycemic parameters, including fasted whole blood glucose, serum insulin, HOMA-IR and plasma glucagon, did not significantly differ at either day 1 or day 43 between the whole grain wheat sourdough and white bread treatments, within either the NGI or HGI group (data not shown, p values not reported).	M
**MacKay et al. 2012 (24)**	Canada	RCT, CO, 6 weeks	14 **normoglycemic** subjects<br>10 males<br>Age: 53 ± 6.0 y<br>BMI :26.5 ± 2.9 kg/m²<br>Whole blood glucose: 4.5 ± 0.40 mmol/L<br><br>14 **hyperglycemic** subjects<br>10 males<br>Age: 57 ± 7.4 y,<br>BMI: 35.7 ± 5.65 kg/m²<br>Whole blood	**Test product:** Wholegrain wheat sourdough bread<br>**Control product:** Refined white wheat bread	Not known (Commercial breads)<br><br>**Authentication:** NM	Not known (Commercial breads)	Not known (Commercial breads)	▪ For postprandial blood glucose, bread treatment by time interaction was not significant within either NGI or HGI.<br>▪ In the HGI group, glucose $iAUC_{0\text{-}180\ min}$ was lower (19%, P < 0.02) after 6-week consumption of whole grain wheat sourdough bread compared to white bread, which may be attributable to a lower (P < 0.01) blood glucose at 2 h post-OGTT after whole grain wheat sourdough bread (6.22 ± 0.50 mmol/L) versus white bread (7.12 ± 0.41 mmol/L) in HGI, but not NGI (whole grain wheat sourdough bread: 4.88 ± 0.33 mmol/L; white bread: 5.42 ± 0.64 mmol/L; P < 0.57).<br>▪ With the exception of a lower (P < 0.05) 3 h blood glucose concentration after whole grain wheat sourdough bread (3.38 ± 0.25 mmol/L) versus white bread (4.02 ± 0.37 mmol/L), there were no differences in blood glucose at any time points in the NGI group.	L

Author/ Year/ Reference	Location	Design	Subjects	Interventions	Strains	Fermentation conditions	Doses in starter (CFU/g flour)	Results	Risk of bias
			glucose: 5.0 ± 0.38 mmol/L						
**Lappi et al. 2014 (21)**	Finland	RCT, CO, 4 weeks	21 **healthy** subjects<br>12 females<br>Age: 38-65 y<br>BMI: 19-30 kg/m²<br>Fasting glucose: 4.9-6.3 mmol/L<br><br>Self-reported gastrointestinal symptoms	**Test product:** sourdough wholegrain rye bread (WGR)<br>**Control product:** white wheat bread enriched with bioprocessed rye bran (BRB + WW) or white wheat bread alone (run-in period only)	**Sourdough:** *L. brevis, L. plantarum,* baker's yeast<br><br>**Control:** baker's yeast<br><br>Strains NM<br><br>**Authentication**: NM	Fermented 22 hours at 30°C, proofed for 50 min in 35°C and 80% RH	NM	**Glucose and insulin responses:**<br>▪ Fasting and postprandial glucose and insulin responses to the meal test did not differ between the WGR and BRB + WW periods. No difference was found in glucose and insulin $AUCs_{0\text{-}120\ min}$.<br>▪ However, response of plasma insulin to the meal test was lower after the WGR period than after the WW period at 120 min (p = 0.023, Wilcoxon test) (Figure 3B).	L
**Najjar et al. 2009 (27)**	Canada	RCT, SB, CO, Acute	10 **overweight or obese** females<br><br>Age: 59.0 ± 2.41 y<br><br>BMI: 30.8 ± 0.95 kg/m²	▪ White Wheat bread with sourdough<br>▪ Whole Wheat bread<br>▪ Whole Wheat / Barley (70:30) bread<br>▪ White Wheat bread	NM ("made from white flour, water and dry yeasts")<br><br>**Authentication**: NM	Breads proofed for 60 min at 30°C (relative humidity 70 %), except for the sourdough which was proofed at room temperature for 3 h	NM	**Glucose response:**<br>▪ There were significant differences in overall glucose responses to the test breads with sourdough being lower than both white (P<0.05) and whole wheat (P<0.007) breads as well as whole wheat barley being lower than whole wheat (P<0.05) bread.<br>▪ For each meal period (0-180 and 180-300 min) as well as entire study (0-300 min), glucose AUC for sourdough was lower than whole wheat (P<0.05) and for both the second meal period and the entire study sourdough was also lower than whole wheat barley (P<0.05) bread.<br>**Blood hormones:**<br>▪ No overall treatment effect was found in GIP responses to the test breads<br>▪ While there were no significant differences for the GIP AUC among the test breads following the first meal, the GIP AUC after sourdough bread ingestion was less than white (P<0.004) and whole wheat barley (P<0.002) breads after the second meal.<br>▪ The GLP-1 response to sourdough was lower than white (P<0.0001) and whole wheat (P<0.0001) breads. In addition, the GLP-1 concentration for white bread was lower than whole wheat barley bread (P<0.02).<br>▪ No significant difference was found in GLP-1 AUC among the	L

Author/ Year/ Reference	Location	Design	Subjects	Interventions	Strains	Fermentation conditions	Doses in starter (CFU/g flour)	Results	Risk of bias
								test breads.	
**Mofidi et al. 2012 (28)**	Canada	RCT, SB, CO, Acute	**Part 1:**<br>12 **overweight** male subjects<br><br>Age: 54.9±2.0 y<br><br>BMI: 29.1±1.1 kg/m²<br><br>Fasting blood glucose: 4.5 ± 0.1 mmol/L<br><br>Fasting serum insulin: 50.8 ± 4.8 pmol/L	**Part 1 --> The test breads were prepared to provide 50 g of available CHO:**<br>▪ 151 g of 11-grain (whole-grain, with sourdough culture),<br>▪ 157 g of sprouted-grain (whole-grain, with sourdough culture)<br>▪ 107 g of sourdough white<br>▪ 122 g of 12-grain (whole-grain)<br>▪ 110 g of white bread	Not known (Commercial breads)<br><br>**Authentication**: NM	Not known (Commercial breads)	Not known (Commercial breads)	<u>**Study part 1 (breads matched for 50 g CHO)**</u><br>**Glucose response:**<br>▪ Sprouted-grain bread $[iAUC]_{0\text{-}180min}$ was significantly lower than 11-grain (P < 0.009), [white] sourdough (P < 0.001), and white (P < 0.006) breads.<br>▪ 12-grain bread $[iAUC]_{0\text{-}180min}$ was significantly lower than 11-grain (P < 0.04) and [white] sourdough (P < 0.003) breads.<br>▪ Similarly, glucose incremental AUC for sprouted grain bread was significantly lower than 11-grain (P < 0.007), [white] sourdough (P < 0.004), and white (P < 0.05) breads<br>▪ Glucose incremental $AUC_{0\text{-}180min}$ for 12-grain bread was significantly lower than 11-grain (P < 0.01) and [white] sourdough (P < 0.009) breads.<br>**Insulin response:**<br>▪ 11-grain bread was higher than [white] sourdough (P < 0.005) and white (P < 0.03) breads<br>▪ Insulin incremental AUC for 11-grain and sprouted-grain breads was significantly (P < 0.05) greater than [white] sourdough and white breads.<br><br>**Blood hormones:**<br>▪ There was no significant overall treatment effect in GIP responses to the breads (data not shown). Similarly, bread treatment did not significantly affect GIP incremental AUC<br>▪ The GLP-1 response to [white] sourdough bread was lower than 11-grain (P < 0.0001), sprouted-grain (P < 0.0001), and white (P < 0.02) breads.<br>▪ GLP-1 response to 11-grain bread was greater than 12-grain (P < 0.03) and white (P < 0.03) breads, while the GLP-1 response to sprouted-grain bread was greater than 12-grain (P < 0.009) and white (P < 0.05) breads.<br>▪ Despite these differences, bread treatment did not significantly affect GLP-1 incremental AUC.	L
**Mofidi et al. 2012 (28)**	Canada	RCT, SB, CO, Acute	**Part 2:**<br><br>11 **overweight** males	**Part 2 -->** The same test breads studied in part 1 were prepared to provide a consistent portion of 107 g	Not known (Commercial breads)	Not known (Commercial breads)	Not known (Commercial breads)	<u>**Study part 2 (breads matched for mass):**</u><br>**Glucose response:**<br>▪ Glucose incremental AUC for [white] sourdough bread was significantly greater than 11-grain (P < 0.002), sprouted-	L

Author/ Year/ Reference	Location	Design	Subjects	Interventions	Strains	Fermentation conditions	Doses in starter (CFU/g flour)	Results	Risk of bias
			Age: 53.9±1.7 y<br><br>BMI: 28.6±0.7 kg/m[2]<br><br>Fasting glucose: 4.6 ± 0.1 mmol/L<br>Fasting insulin:40.6 ± 5.7 pmol/L		**Authentication**: NM			grain ($P < 0.01$), 12-grain ($P < 0.001$), and white ($P < 0.04$) breads.<br>**Insulin response:**<br>▪ Sprouted-grain lower than 12-grain ($P < 0.03$) bread and 12-grain bread being lower than [white] sourdough ($P < 0.001$) and white ($P < 0.001$) breads.<br>▪ Insulin incremental AUC for 11-grain ($P < 0.03$), sprouted-grain ($P < 0.05$), and 12-grain ($P < 0.0007$) breads was significantly lower than [white] sourdough bread<br>▪ Insulin incremental AUC for 12-grain was lower than white bread ($P < 0.03$).<br><br>**Blood hormones:**<br>▪ Overall GIP response to 11-grain was lower than [white] sourdough bread ($P < 0.008$).<br>▪ GIP incremental AUC for 11-grain bread was significantly lower than [white] sourdough ($P < 0.03$) and white ($P < 0.001$) breads.<br>▪ GLP-1 response did not relate to the amount of available CHO consumed as the overall GLP-1 response to sprouted-grain bread was significantly greater than 11-grain ($P < 0.008$), [white] sourdough ($P < 0.001$), 12-grain ($P < 0.04$), and white ($P < 0.04$) breads.<br>▪ GLP-1 incremental AUC for sprouted-grain was significantly greater than [white] sourdough ($P < 0.05$) and 12-grain ($P < 0.01$) breads.	
**Tucker et al. 2014 (25)**	Canada	RCT, CO, Acute	12 **type-2 diabetes (T2D)** subjects<br><br>11 males, 1 female<br>Age: 63.8 ± 3.30 y<br>BMI: 32.5 ± 1.41 kg/m²<br>HbA1c: 6.94 ± 0.41%<br>Fasting blood glucose: 6.88 ± 0.58 mmol/L	**Treatment breads:**<br>▪ Sprouted sourdough (wheat; SPR),<br>▪ Whole-grain sourdough (mainly wheat; WG),<br>▪ Enriched white sourdough (wheat flour enriched with added lactic acid, ascorbic acid and sodium acetate; SD),<br>▪ Enriched white (same as SD without sourdough; WB).	Not known (Commercial breads)<br><br>**Authentication**: NM	Not known (Commercial breads)	Not known (Commercial breads)	▪ Postprandial blood glucose after the bread meal did not significantly differ between the bread treatments; however, at 15 min after the second meal (195 min), it was significantly lower with consumption of WG compared to SD, but not SPR or WB.<br>▪ Glucose iAUC after the bread meal and total time was significantly lower with consumption of SPR compared to WG, SD and WB, but did not significantly differ after the second meal among bread treatments.	L

Author/ Year/ Reference	Location	Design	Subjects	Interventions	Strains	Fermentation conditions	Doses in starter (CFU/g flour)	Results	Risk of bias
**Fredensborg et al. 2010 (49)**	New Zealand	RCT, Acute	30 **healthy subjects**<br><br>19 females<br>Age (y): 33.7 ± 14.3, 30.4 ± 9.1, 27.4 ± 6.6 (groups 1, 2, 3 resp.)<br>BMI (kg/m²): 23.9 ± 2.6, 22.8 ± 3.0, 22.7 ± 2.6 (groups 1, 2, 3 resp.)<br>Fasting glucose (mmol/L): 5.0 ± 0.5, 4.7 ± 0.2, 4.4 ± 0.2 (groups 1, 2, 3 resp.)	▪ **Group 1:** short rye, short oat and long whole meal (whole wheat)<br>▪ **Group 2:** long rye, long oat and short whole meal<br>▪ **Group 3:** Yeast, Desem, Sourdough, Sourdough + oats, and Swiss Rye™	**Sourdough**: NM<br><br>**Control:** Baker's yeast<br><br>**Authentication**: NM	**Sourdough:** 80 min rising, 60 min proving, 60 min baking at 200°C	NM<br><br>Sourdough starter 70% wet weight	**Glycemic index:**<br>▪ Regression analysis revealed significant differences between the GI of Swiss Rye™ and Yeast (p = 0.010), Swiss Rye™ and Desem (p= 0.007) and Sourdough+oats and Desem (p = 0.043).<br>▪ Mean plasma glucose peaked at 30 minutes following ingestion of eight of the breads, and at 45 minutes for the remaining three breads (Long rye, Short rye and Long whole meal) (Figure 1).<br>▪ However, mean plasma glucose concentration had not returned to baseline on completion of testing at 120 minutes for any of the breads tested.	L
**Korem et al. 2017 (22)**	Israel	RCT, 1 week	20 **heathy** subjects<br>11 females<br><br>**In test group:**<br>Age: 39.1 ± 14.3 y<br>BMI 26.5 ± 5.6 kg/m²<br>Total cholesterol: 191 ± 40 mg/dL<br><br>**In control group:**<br>Age: 37.3 ± 8.9 y<br>BMI: 24.6 ± 5.3 kg/m²<br>Total cholesterol: 171 ± 26 mg/dL	**Test product:** Whole wheat sourdough bread<br>**Control product:** White wheat bread	NM ("traditional method")<br><br>**Authentication**: NM	NM	NM	We found no significant difference between the two treatments both for the primary outcome measure of this trial, glycemic control, which we quantified using the response to an oral glucose tolerance test (OGTT; Figures 1B and 1C) and wake-up glucose levels (Figures 1B and 1D), and for 18 secondary outcome measures (Figures 1B and S2).	L
**Juntunen et al. 2003 (16)**	Denmark	RCT, CO, Acute	19 healthy post-menopausal females<br><br>Age: 61 ± 1 y<br>BMI: 26.0 ± 0.6 kg/m²	**Breads providing each 50 g available CHO:**<br>**Control bread:**<br>▪ Commercial refined wheat bread<br>▪ Endosperm rye bread<br>▪ Traditional rye bread<br>▪ High-fiber rye bread	**Sourdough:**<br>▪ L62 (0.4 g *L. brevis*)<br>▪ L73 (0.4 g *L. plantarum*),<br>▪ fresh yeast	NM	NM	▪ The maximal glucose responses, the times to reach the maximal response, and the AUCs did not differ significantly among the breads.<br>▪ However, glucose concentrations in response to refined wheat bread had fallen below baseline fasting concentrations and were lower than corresponding concentrations in response to endosperm rye bread and to	M

Author/ Year/ Reference	Location	Design	Subjects	Interventions	Strains	Fermentation conditions	Doses in starter (CFU/g flour)	Results	Risk of bias
			with normal glucose tolerance		(3.8 g)<br><br>**Authentication**: NM			traditional rye bread at 150 and 180 min (P = 0.012–0.036) and to high-fiber rye bread at 180 min (P = 0.048).<br>▪ The plasma GIP responses to rye breads were significantly lower than those to wheat bread at several time points:<br>- For endosperm rye bread at 60 and 90 min (P = 0.0001 and P = 0.018, respectively);<br>- for traditional rye bread at 30, 45, 60, 90, and 120 min (P = 0.0001–0.006);<br>- and for high-fiber rye bread at 30, 45, 60, and 90 min (P = 0.0001–0.006).	
**Dall'Asta et al. 2022 (14)**	Italy	RCT, CO	14 healthy volunteers aged 24 ± 3 y	**7 breads made from 2 bread wheat evolutionary populations (Bio2 and ICARDA) or a modern bread wheat variety (Bologna) (all type 1 flour *i.e.* whole meal except control):**<br>▪ Bologna control (refined flour) with yeast<br>▪ Yeast Bologna<br>▪ Sourdough Bologna<br>▪ Bio2 yeast<br>▪ Bio2 sourdough<br>▪ ICARDA yeast<br>▪ ICARDA sourdough	Not known (prepared by the professional baker)<br><br>**Authentication**: NM	Leavened in a prover for 2 days at 30°C, 66% RH<br><br>Bulk fermentation at 28°C, 66% RH for 60 min (yeast bread) or 90 min (sourdough bread)	NA	**Postprandial blood glucose responses**<br>▪ A main effect of both the time (P < 0.001) and leavening (P < 0.001), but not for the type of flour and the interaction of these factors, was observed by the comparison of the postprandial glycemic response (PPGR).<br>▪ The iAUC values for glucose ranged from 124.8 ± 14.2 mmol/L/ min (EP Bio2 S) to 183.7 ± 16.0 mmol/L/min (Bologna Y), which was, interestingly, the only sample rich in fiber (> 6 g/100 g)<br>▪ However, statistical comparison among the breads administered to participants showed no effect on iAUCs induced either by the type of flour or by the leavening technique (common yeast or sourdough) used for baking, or the interaction between these 2 factors (flour x leavening).<br>**Hunger and satiety ratings**<br>▪ After statistical comparison of iAUCs, no differences among samples were found for both hunger and satiety feelings	M

Abbreviations: AUC: Area Under the Curve; BMI: Body Mass Index; CFU: Colony Forming Unit; CHO: Carbohydrates; CO: Crossover; CS: Celiac sprue; DB: Double-Blind; GCV: Gastric Content Volume; GI: Gastrointestinal Index; HbA1c: glycated hemoglobin; iAUC: incremental Area Under the Curve; IBS-SS: Irritable Bowel Severity Scoring System; NM: not mentioned; RCT: Randomized Controlled Trial; SB: Single-Blind; TGV: Total Gastric Volume; VAS: Visual analogue Scale.

.

Characteristics of clinical trials investigating the effects of sourdough bread on **appetite markers**, either as primary or secondary outcome.

Author/ Year/ Reference	Location	Design	Subjects	Interventions	Strains	Fermentation conditions	Doses in starter (CFU/g flour)	Results	Risk of bias
**Liljeberg et al. 1995 (11)**	Sweden	RCT Acute	11 **healthy** subjects<br><br>Age: 26-48 y	Whole-meal bread (WMB: Barley/Wheat 80:20, with yeast) alone or with added:<br>▪ Sourdough<br>▪ Lactic acid (64 g)<br>▪ Ca-lactate (95 g)<br>▪ Na-propionate (62 g)<br>▪ Na-propionate, high [] (185 g)	*L. plantarum* A1 (ClasLönner AB, Lund, Sweden)<br><br>**Authentication**: NM	Proofed 50 min + 20 min, 38°C, 75% humidity	5.10^{+7} CFU/g flour	**Satiety scores:**<br>▪ In the early phase, 45 min after the ingested meal, the two bread products with added Na-propionate were given higher satiety scores ($P < 0.05$) than the whole-meal bread (WMB) product. For the bread plus high Na-propionate, this was also true at 95 min.<br>▪ In addition, at 120 min, significantly higher satiety scores were registered with WMB plus Ca-lactate and WMB plus Na-propionate, compared with WMB ($p < 0.05$).<br>▪ When calculating the satiety area under the curves (0-180 min), a significantly higher value was found with the bread baked with the high concentration of Na-propionate than with WMB ($p < 0.05$).	M
**Darzi et al. 2012 (15)**	United Kingdom	RCT SB CO Acute	20 **healthy** subjects<br><br>Age: 25.1 ± 4.6 y	White wheat-bread sandwich administered in breakfast:<br>▪ Propionate-rich sourdough bread (2.3 mg per g propionic acid and 2.98 mg per g calcium propionate)<br>▪ Control bread	"Domani starter culture"<br><br>NM<br><br>**Authentication**: NM	NM	NM	**Ad libitum energy intake:**<br>▪ The mean EI of the ad libitum pasta test lunch provided 180 min postprandially did not differ between treatments following consumption of control and SOUR breakfast meals (p not mentioned)<br>▪ The 24 h mean EI for the entire study day was 543 ± 2070 kJ lower with SOUR treatment than with control. However, this difference was non-significant and no significant differences in macronutrient intake were found between treatments (p not mentioned)<br>**Appetite ratings:**<br>▪ No treatment or treatment x time effects were found for postprandial appetite ratings over 180 min for fullness, hunger, prospective consumption or desire to eat (p not mentioned)<br>▪ Postprandial ratings for the desire to eat something sweet were significantly lower ($P=0.024$) following SOUR than following control	H

Author/ Year/ Reference	Location	Design	Subjects	Interventions	Strains	Fermentation conditions	Doses in starter (CFU/g flour)	Results	Risk of bias
**Rizzello et al. 2019 (13)**	Italy	RCT	36 **healthy** subjects,<br><br>Age :25 ± 1.1 y	**Three types of refined wheat bread:**<br>▪ Traditional sourdough bread (t-SB) (*i.e.* without baker's yeast)<br>▪ Sourdough bread (SB) (LAB + baker's yeast)<br>▪ Baker's yeast bread (BYB)	*L. plantarum CR1, L. rossiae CR5, S. cerevisiae E10.*<br><br>**Authentication**: Culture Collection of the Department of Soil, Plant and Food Sciences (University of Bari, Italy)	4 h at 30 °C (step I)<br><br>1.5 h at 30 °C (step II)	5.10^{+7} (lactic acid bacteria)<br><br>5.10^{+6} (yeast)	**Appetite & Satiety:**<br>▪ Using VAS approach, appetite and satiety, and the gastrointestinal symptoms were monitored during 120 min following ingestion.<br>▪ After 30 min, SB and t-SB stimulated more appetite than BYB (p values not mentioned)<br>▪ The appetite AUC did not differ significantly ($p > 0.05$) between BYB and t-SB, but was significantly higher ($p < 0.05$) in the SB group *vs.* the BYB group<br>▪ The satiety AUC did not differ significantly ($p > 0.05$) between BYB and t-SB, but was significantly lower ($p < 0.05$) in the SB group *vs.* the BYB group	L
**Bo et al. 2017 (19)**	Italy	RCT DB CO Acute	16 **healthy** subjects<br><br>6 males, 10 females<br>Age: 32.3 ± 7.4 y	**Test product(s):** Sourdough bread made with commercial wheat flour, organic wheat flour or organic einkorn flour<br>**Control product(s):** Bread made with commercial wheat flour, no sourdough	**Sourdough:** *L. acidophilus* (strain NM) *L. casei* (strain NM) *S. cerevisiae* (strain NM) *S. exiguous* (strain NM)<br><br>**Control:** NM<br><br>**Authentication**: NM	4-5 h at 28°C and 40% of relative humidity	50 g of starter culture (9.510^{+5}CFU/g flour)	**Blood hormones & satiety:**<br>▪ Ghrelin AUC was significantly lower and satiety significantly higher after consuming the einkorn bread with respect to the commercial wheat bread ($p = 0.004$), but not with the other sourdough breads ($p = 0.92$ for sourdough commercial wheat bread and $p= 0.23$ for organic wheat bread, compared to non-sourdough commercial bread).	L
**Zamaratskaia et al. 2017 (18)**	Sweden	RCT SB CO Acute	24 **healthy** subjects<br><br>Age: 30 ± 11 y	▪ Sourdough-fermented whole grain (19 %) rye crispbread<br>▪ unfermented whole grain rye crispbread, and<br>▪ refined wheat crispbread (control)	**Sourdough:**« in-house culture mixture » (spontaneous)<br><br>**Control:** yeast<br><br>**Authentication**: NM	Two-step fermentation: first for 120 min at 29°C, followed by 35 min with an increase from 30 to 38°C.	NM	**Subjective appetite ratings:**<br>▪ Significant overall effects of diet for hunger and desire to eat (P=0.03 for both) were observed when comparing AUC (0–360 min), but diet did not affect these parameters when modelling the curves (P=0.07 and 0.09, respectively) (Fig. 2).<br>▪ The differences in AUC for hunger were because of lower scores after sourdough-fermented rye crispbread vs. yeast-fermented refined wheat crispbread, whereas the difference in desire to eat was because of lower scores for both rye	L

Author/ Year/ Reference	Location	Design	Subjects	Interventions	Strains	Fermentation conditions	Doses in starter (CFU/g flour)	Results	Risk of bias
								breads vs. yeast-fermented refined wheat crispbread (Fig. 2, right).<br>▪ The main effect on fullness did not reach statistical significance for AUC (P= 0.08) or for curves (P=0.06) (Fig. 2, left).	
**Iversen et al. 2018 (29)**	Sweden	RCT CO Acute	23 **healthy** subjects<br>15 females, 8 males<br><br>Age: 32 ± 9.8 y<br><br>BMI: 22.5 ± 2.7 kg/m²	**Test products:**<br>5 whole rye/wheat breads:<br>▪ MS/MR = Medium Sourdough (30%) / Medium Rye (42%)<br>▪ HS/LR = High Sourdough (51%) / Low Rye (35%)<br>▪ HS/HR = High Sourdough (51%) / High Rye (48%)<br>▪ LS/LR = Low Sourdough (9%) / Low Rye (35%)<br>▪ LS/HR = Low Sourdough (9%) / High Rye (48%)<br><br>**Control product**<br>▪ Refined wheat bread	**Sourdough:**<br>*Lactobacillus brevis A6* (18 g)<br>*S. cerevisiae TY08M* (10 g)<br><br>**Control:** yeast<br><br>**Authentication**: NM	Incubated at 30°C for 24 h before use in the baking. Fermented 38°C, 75% RH for 60 min	NM	**Appetite ratings:**<br>▪ Pairwise comparisons showed that medium sourdough/medium rye (MS/MR), high sourdough/high rye (HS/HR), and low sourdough/high rye (LS/HR) resulted in higher fullness rating, compared to the reference white wheat bread<br>▪ MS/MR and LS/HR resulted in lower hunger ratings, compared to refined bread, according to model B (*i.e.* AUC data was analyzed in an ANCOVA model), while there was only a tendency according to model A (i.e. within subject repeated measures ANCOVA) (Figure 3). Excluding the two subjects with BMI outside the desired range did not alter the conclusions with regards to hunger and fullness, but reduced the effect of diet on desire to eat to a tendency (model A: p = 0.051; model B: p = 0.057)<br>▪ No significant differences were observed when comparing the test breads<br>**Ad libitum energy intake:**<br>▪ Analysis of the amount of energy consumed in the ad libitum lunch test revealed an effect of diet (p = 0.032), with a 0.5 MJ reduction in energy intake following consumption of LS/LR and LS/HR at breakfast, compared to the reference bread (Figure 4). Removing the two subjects with BMI outside the desired range from the analysis, reduced the effect on energy intake following LS/HR and it was no longer different from the reference bread (mean difference 0.3 MJ, p = 0.319)<br>▪ Analysis of the factorial design revealed no effect of rye or sourdough on appetite response.	L
**Polese et al. 2018 (30)**	Italy	RCT DB CO Acute	17 **healthy** subjects<br><br>9 females<br><br>BMI: 22.4 ± 2.2 kg/m²	**Test product:** Sourdough croissant<br><br>**Control product:** Baker's yeast croissant	**Sourdough:**<br>*Lactobacillus sanfranciscensis* (about 85%), *Streptococcus sp., Leuconostoc sp.*	NM	NM	**Appetite ratings:**<br>▪ Moreover, the AUC analysis of gastrointestinal perceptions showed that the feeling of hunger was significantly lower from 45 to 240 min after consuming the SCs compared with after the BCs (P = 0.01; Figure 4B). In the same time interval, even appetite tended to be lower after intake of the SCs than after the BCs (P = 0.09; Figure 4B).	L

Author/ Year/ Reference	Location	Design	Subjects	Interventions	Strains	Fermentation conditions	Doses in starter (CFU/g flour)	Results	Risk of bias
			Blood glucose 75.6 ± 6.3 mg/dL		(lower %)<br><br>**Control:** Baker's yeast (*S. cerevisiae*)<br><br>**Authentication**: culture-independent amplicon-sequencing approach based on the taxonomically relevant 16S ribosomal RNA gene			▪ The feeling of satiety was significantly greater from 45 to 240 min after consuming the SCs than after the BCs (P = 0.03; Figure 4B).	

Abbreviations: AUC: Area Under the Curve; BMI: Body Mass Index; CFU: Colony Forming Unit; CHO: Carbohydrates; CO: Crossover; CS: Celiac sprue; DB: Double-Blind; GCV: Gastric Content Volume; GI: Gastrointestinal Index; HbA1c: glycated hemoglobin; iAUC: incremental Area Under the Curve; IBS-SS: Irritable Bowel Severity Scoring System; NM: not mentioned; RCT: Randomized Controlled Trial; SB: Single-Blind; TGV: Total Gastric Volume; VAS: Visual analogue Scale.

<u>Characteristics of clinical trials investigating the effects of sourdough bread on **gastrointestinal markers**, either as primary or secondary outcome.</u>

Author/ Year/ Reference	Location	Design	Subjects	Interventions	Strains	Fermentation conditions	Doses in starter (CFU/g flour)	Results	Risk of bias
**Polese et al. 2018 (30)**	Italy	RCT, DB, CO, Acute	17 **healthy** subjects<br><br>9 females<br><br>BMI: 22.4 ± 2.2 kg/m²<br><br>Blood glucose 75.6 ± 6.3 mg/dL	**Test product:** Sourdough croissant<br>**Control product:** Baker's yeast croissant	**Sourdough:** *Lactobacillus sanfranciscensis* (about 85%), *Streptococcus sp.*, *Leuconostoc sp.* (lower %)<br><br>**Control:** Baker's yeast (*S. cerevisiae*)<br><br>**Authentication**: culture-independent amplicon-sequencing approach based on the taxonomically relevant 16S ribosomal RNA gene	NM	NM	**Gastric emptying:**<br>▪ The assessment of gastric kinetic curves with the ANOVA showed no significant difference (P = 0.55) after SC and BC ingestion in both Total Gastric Volume (TGV) and Gastric Content Volume (GCV) curves.<br>▪ The AUC analysis showed that Total Gastric Volume was significantly lower from 0 to 180 min after consuming the SCs than after consuming the BCs (P = 0.02).<br>▪ In the same time interval, the decrease in Gastric Content Volume after intake of the SCs (559 ± 37.6 dL/min) tended to be lower than after intake of the BCs (618 ± 24.4 dL/min) (P = 0.07).<br>**Gastric fermentation:**<br>▪ The AUC analysis of hydrogen concentration in end expiratory air for the assessment of gastrointestinal fermentation showed that hydrogen production from 45 to 240 min after the ingestion of the SCs was significantly lower compared with after the BCs (P = 0.03).<br>▪ The evaluation of hydrogen production kinetics by ANOVA showed no significant difference (P = 0.99).<br>**Gastrointestinal symptoms:**<br>▪ The AUC analysis of VASs showed that from 0 to 240 min after the ingestion of the SCs, the overall severity of gastrointestinal symptoms was slightly reduced when compared with after the BCs (P = 0.05).<br>▪ Specifically, participants reported significantly lower abdominal discomfort (P = 0.002; Figure 4A), bloating (P = 0.001; Figure 4A), and nausea (P = 0.004; Figure 4A) after intake of the SCs than after the BCs.<br>▪ Conversely, there was no significant difference in terms of fullness of either SCs or BCs (P = 0.36; Figure 4A).	**L**

Author/ Year/ Reference	Location	Design	Subjects	Interventions	Strains	Fermentation conditions	Doses in starter (CFU/g flour)	Results	Risk of bias
**Rizzello et al. 2019 (13)**	Italy	RCT	36 **healthy** subjects<br><br>Age: 25 ± 1.1 y	**Three types of refined wheat bread:**<br>▪ Traditional sourdough bread (t-SB) (*i.e.,* without baker's yeast)<br>▪ Sourdough bread (SB) (LAB + baker's yeast)<br>▪ Baker's yeast bread (BYB)	*Lb. plantarum CR1, Lb. rossiae CR5 and S. cerevisiae E10*<br><br>**Authentication**: Culture Collection of the Department of Soil, Plant and Food Sciences (University of Bari, Italy)	4 h at 30 °C (step I)<br>1.5 h at 30 °C (step II)	5.10^{+7} (lactic acid bacteria)<br><br>5.10^{+6} (yeast)	**GI transit:**<br>▪ Volunteers showed similar fasting gallbladder volumes across the four days of challenging (17.6 ± 1.2 −18.9 ± 1.3 mL).<br>▪ Compared to breads, NU (Nutridrink used as the reference) induced a faster gallbladder emptying and refilling.<br>▪ The emptying rate corresponding to SB and t-SB ingestion was significantly ($p < 0.05$) lower than that of BYB (−0.24 ± 01 and −0.28 ± 0.1 vs. −1.20 ± 0.1 cm2/min. Accordingly, the half-emptying time of BYB (43.7 ± 4.4 min) was significantly ($p < 0.05$) longer than that found for SB and t-SB (34.8 ± 2.4 and 30.8 ± 2.3 min, respectively). The AUC amplitude for BYB was the highest (854 ± 119 vs. 774 ± 105 and 758 ± 105 cm2 × 120 min for SB and t-SB, respectively).<br>▪ The OCTT median value for BYB was 89.5 min, which was not significantly ($p > 0.05$) different than that found for SB (80.5 min). On the contrary, this value was markedly and significantly ($p < 0.05$) longer than that of t-SB (69.5 min).<br>**GI symptoms:**<br>▪ No symptoms of nausea were perceived (scores always lower than 10 mm) after ingesting breads (Figure 2B). Significantly ($p < 0.05$) higher scores were reported after NU ingestion.<br>▪ The consumption of NU also associated to the lowest fullness perception. AUC for fullness were similar among breads. Nevertheless, t-SB showed the highest score at 30 min<br>▪ Volunteers did not report epigastric pain after ingesting breads or NU. In particular, VAS scores were lower than 15 mm during the 120 min following the samples ingestion. AUC was similar ($p > 0.05$) and lower than 1000 for all the breads and NU.	**L**

Author/ Year/ Reference	Location	Design	Subjects	Interventions	Strains	Fermentation conditions	Doses in starter (CFU/g flour)	Results	Risk of bias
**Laatikainen et al. 2016 (31)**	Finland	RCT, DB, CO, 4weeks	87 **Irritable Bowel Syndrome (IBS)** subjects<br><br>91.3 % females<br><br>Age: 42.9 (21–64) y<br><br>Median BMI: 23.4 (17.3–36.6) kg/m^2	**Two rye / wheat sourdough breads:**<br>▪ Traditional rye sourdough bread (1.1 g/100 g fructans)<br>▪ Low-FODMAP rye sourdough bread (0.3 g/100 g fructans)	NM<br><br>Authentication: NM	NM	NM	**IBS-SSS:**<br>▪ Mean IBS-SSS score was 228 (range 80–430) at baseline. Most patients (62.5%) had mixed type of IBS, 32.5% had diarrhœa predominant IBS and 5% had unspecified symptoms.<br>▪ There was no significant difference in IBS-SSS values between the study breads. The estimated mean was 199 (95% CI 179–220) during the low-FODMAP rye bread vs. 207 (187–227) during the regular rye bread. The difference between breads was 8 (95% CI 27–11), P = 0.40<br>**Quality of life:**<br>▪ No significant difference was detected between the study breads in the assessment of quality of life. The mean (95% CI) was 29.2 (25.0–33.5) during the low-FODMAP rye bread vs. 30.0 (25.5–34.5) during the regular rye bread. The difference was 0.8 (2.9–1.3), P = 0.45.<br>**Abdominal symptoms (VAS):**<br>▪ Weekly VAS measurements of individual symptoms revealed statistically significant differences between the breads when the mean values of weeks 1, 2, 3 and 4 were analysed (Table 4).<br>▪ Flatulence, abdominal pain, cramps and stomach rumbling were milder when the subjects were eating the low-FODMAP rye bread (P-values: 0.04; 0.049; 0.01 and 0.001 respectively).<br>▪ There was also a significant difference between the breads in the total symptom score, i.e. in the mean of all 10 symptoms, 30 (95% CI 27–30) during the low-FODMAP rye bread vs. 33 (95% CI 30–37) during the regular rye bread. The mean difference was -3 (-6 to -1), P = 0.02) favouring low-FODMAP rye bread.<br>▪ The breath hydrogen level was significantly lower during the low-FODMAP rye bread as compared to the regular rye bread. The median (inter-quartile range) AUC was 52.9 (32.4–76.8) ppm during low-FODMAP rye bread vs. 72.6 (46.7–114.9) ppm during the regular rye bread (P = 0.01, Wilcoxon signed-rank test).	**L**

Author/ Year/ Reference	Location	Design	Subjects	Interventions	Strains	Fermentation conditions	Doses in starter (CFU/g flour)	Results	Risk of bias
**Laatikainen et al. 2017 (32)**	Finland	RCT, DB, 1 week	26 **Irritable Bowel Syndrome (IBS)** subjects<br><br>96% females<br>Age: 43 (21-64) y<br>BMI: 25.0 (19.6-37.7) kg/m²	**Test product:**<br>Low-FODMAPS refined wheat sourdough breads (fructans: 0.06 g/100 g)<br><br>**Control product:**<br>Refined wheat yeast bread (fructans: 0.23 g/100 g)	NM<br><br>**Authentication**: NM	NM	NM	**GI symptoms (VAS):**<br>▪ VAS measurements of gastrointestinal symptoms did not reveal any statistically significant differences between the breads, when the weekly means were analysed.<br>▪ There was no significant difference between the breads in terms of the total gastrointestinal symptom score (i.e., the mean of 12 symptoms) which was 27 (Standard deviation, SD 12) mm for the sourdough bread vs. 23 (SD 11) mm for the yeast-fermented bread<br>**Non-GI symptoms:**<br>▪ There was a significant difference between the breads for the total non-gastrointestinal score, i.e., in other symptoms, the mean of five symptoms was 26 (SD 18) mm for the sourdough bread vs. 11 (SD 10) mm for the yeast-fermented bread<br>▪ More specifically, tiredness, joint symptoms, and decreased alertness were more intense when the subjects were eating the sourdough bread (Table 6, p-values: 0.01, 0.03 and 0.003, respectively).<br>**Inflammatory markers:**<br>▪ We did not find significant differences between the study breads in the inflammatory markers (Interleukin-8 (IL-8; pg/mL), interleukin-6 (IL-6; pg/mL) and lipopolysaccharide binding protein (LBP; µg/mL)) (Table 4)<br>▪ Furthermore, the changes in inflammatory markers from baseline to the end of treatment period were not significant	L
**Calasso et al. 2018 (33)**	Italy	RCT, DB, CO, 2 weeks	20 **Irritable Bowel Syndrome (IBS)** subjects<br><br>12 females, 8 males<br><br>Age: 19-66 y	**Two types of bread:**<br>▪ Sourdough wheat bread with reduced-gluten content (50% gluten reduction RG Bread)<br>▪ Baker's yeast wheat bread with normal-gluten content (NG Bread)<br><br>**Two types of pasta:**<br>▪ Sourdough wheat pasta with reduced-gluten content (RG Pasta)<br>▪ Baker's yeast wheat pasta with normal-gluten content (NG Pasta)	**Sourdough:**<br>*L. sanfranciscensis* 7A, LS3, LS10, LS19, LS23, LS38 and LS47, *L. alimentarius* 15M, *L. brevis* 14G, and *L. hilgardii*51B + fungal proteases<br><br>**Control:**	30°C for 1.5 h	8 log cfu/g	**Gluten content:**<br>As determined by R5-ELISA, the untreated *T. durum* and *T. aestivum* wheat flours used for sourdough fermentation contained ca. 75,000 ± 1650 and 74,060 ± 814 ppm of immune reactive gluten, respectively, *versus* 35,960 ± 1550 ppm and 38,650 ± 810 respectively in the hydrolyzed flours, after 8h of fermentation.<br><br>**Habitual diet (baseline) versus gluten-free diet:**<br>▪ Compared to baseline, IBS-SS and VAS significantly decreased after two weeks of run-in open GFD (237 ± 70.25 vs. 164.8 ± 79.15; p < 0.001, and 4.0 ± 1.54 vs. and 2.8 ± 1.40; p < 0.001 respectively)<br>▪ HDAS and IBS-QoL decreased from 22.9 ± 12.70 to 20.3 ±	L

Author/ Year/ Reference	Location	Design	Subjects	Interventions	Strains	Fermentation conditions	Doses in starter (CFU/g flour)	Results	Risk of bias
					Saccharomyces cerevisiae<br><br>**Authentication**: NM			10.65 ($p < 0.028$) and from 50.8 ± 27.94 to 39.5 ± 24.92 ($p = 0.000$) respectively<br><br>**Reduced-gluten diet versus normal-gluten diet:**<br>▪ Compared to normal-gluten, the administration of a reduced-gluten diet resulted in a decrease of the VAS score ($p = 0.042$), while no differences were found in the HDAS and IBS-QoL<br>▪ Ten patients (50%) had and improvement of at least 30% of the VAS and/or IBS-SS and defined as responders. Responders were significantly younger (31.28 ± 1.5 vs 38.3 ± 2.5; $p = 0.046$) and less severe compared to non-responders<br>▪ Permutation analysis showed the highest similarity between the open gluten-free and the reduced-gluten content diet versus the habitual diet and normal-gluten content diet (Figure 4), irrespective of the responder's status. No adverse events related to the study product were reported.	
**Bondia-Pons et al. 2011 (20)**	Finland	Non-randomized study, Acute	16 **healthy** 13 females, 3 males<br><br>Age: 23 ± 3.7 y	**Test product:** Rye sourdough bread (refined) (RB)<br>**Control product:** White wheat bread (WB)	Not known (Commercial breads)<br><br>**Authentication**: NM	Not known (Commercial breads)	Not known (Commercial breads)	**Gastric emptying rate (GER)**<br>▪ No significant differences were observed between the test breads regarding the GER parameters (RB vs. WB: t lag (min) = 97 ± 20 vs. 104 ± 28; t ½ (min) = 151 ± 50 vs. 149 ± 39) (p value not mentioned).	H
**Lappi et al. 2014 (21)**	Finland	RCT, CO, 4 weeks	21 **healthy** subjects<br><br>Age: 38-65 y<br>BMI: 19-30 kg/m²<br>Fasting glucose: 4.9-6.3 mmol/L<br><br>Self-reported gastrointestinal symptoms	**Test product:** sourdough wholegrain rye bread (WGR)<br><br>**Control product:** white wheat bread enriched with bioprocessed rye bran (BRB + WW) or white wheat bread alone (run-in period only)	**Sourdough:** *L. brevis, L. plantarum,* baker's yeast<br><br>**Control:** baker's yeast<br><br>Strains: NM<br><br>**Authentication**: NM	Fermented 22 hours at 30°C, proofed for 50 min in 35°C and 80% RH	NM	**Abdominal comfort:**<br>▪ The gastrointestinal quality of life of the subjects was significantly better ($p < 0.01$) over the 4-week run-in and test periods than at the screening when they followed their habitual diet<br>▪ The subjects reported slight to moderate flatulence more frequently over the BRB + WW and WGR periods than over the WW period ($p < 0.05$).<br>▪ There were no reported differences in bloating, rumbling of stomach, abdominal pain, or heartburn among the periods<br>▪ Frequency of defecation was not affected by the period, being on average 1.4 times per day	L

Abbreviations AUC: Area Under the Curve; BMI: Body Mass Index; CFU: Colony Forming Unit; CHO: Carbohydrates; CO: Crossover; CS: Celiac sprue; DB: Double-Blind; GCV: Gastric Content Volume; GI: Gastrointestinal Index; HbA1c: glycated hemoglobin; iAUC: incremental Area Under the Curve; IBS-SS: Irritable Bowel Severity Scoring System; NM: not mentioned; RCT: Randomized Controlled Trial; SB: Single-Blind; TGV: Total Gastric Volume; VAS: Visual analogue Scale.

Characteristics of clinical trials investigating the effects of sourdough bread on **cardiovascular markers**, either as primary or secondary outcome.

Author/ Year/ Reference	Location	Design	Subjects	Interventions	Strains	Fermentation conditions	Doses in starter (CFU/g flour)	Results	Risk of bias
**MacKay et al. 2012 (24)**	Canada	RCT CO 6 weeks	14 **normoglycemic** subjects<br><br>10 males<br><br>Age: 53 ± 6.0 y<br><br>BMI 26.5 ± 2.9 kg/m²<br><br>Whole blood glucose: 4.5 ± 0.40 mmol/L<br><br>14 **hyperglycemic** subjects<br>10 males<br><br>Age: 57 ± 7.4 y<br><br>BMI: 35.7 ± 5.65 kg/m²<br><br>Whole blood glucose: 5.0 ± 0.38 mmol/L	**Test product:** Wholegrain wheat sourdough bread<br><br>**Control product:** Refined white wheat bread	Not known (Commercial breads)<br><br>**Authentication**: NM	Not known (Commercial breads)	Not known (Commercial breads)	**Fasting PAI-1 and tPA:**<br>▪ Fasting PAI-1 antigen and activity did not differ between the whole grain wheat sourdough and white bread treatments, within either NGI (p = 0.84 and p = 0.50 respectively) or HGI (p = 0.57 and p = 0.98 respectively) (Table 3).<br>▪ Similarly, fasting tPA antigen and activity did not differ between bread treatments, within either NGI (p = 0.49 and p = 0.94 respectively) or HGI (p = 1.00 and p = 0.39 respectively) (Table 3).	L

Author/ Year/ Reference	Location	Design	Subjects	Interventions	Strains	Fermentation conditions	Doses in starter (CFU/g flour)	Results	Risk of bias
**Pagliai et al. 2020 (34)**	Italy	RCT DB CO<br>4 weeks	17 **healthy** subjects<br><br>7 females 10 males<br><br>Age: 34.6 ± 9.1 y	▪ Group A: bread made with ancient grain "Verna" and sourdough (SD),<br>▪ Group B: control bread, made with ancient grain "Verna" and baker's yeast (BY)	**Sourdough:** *Lactobacillus sanfranciscensis, Lactobacillus plantarum, Saccharomyces cerevisiae*<br><br>**Control:** *Saccharomyces cerevisiae*<br><br>**Authentication**: genotypic identification (ARDRA)	NM	2.64E+08 (LAB)<br><br>5.75 E+06 (yeast)	**Blood lipids:**<br>▪ Statistically significant reduction of LDL cholesterol after both SD and BY *versus* baseline. In particular, a reduction of 10.6% (p = .025) and 8.53% (p = .047) was observed after SD and BY, respectively. No significant changes to baseline were reported for triglycerides, total cholesterol, HDL cholesterol, and uric acid in both groups.<br>▪ When comparing statistically SD to BY in terms of change to baseline, no significant differences were identified in any of the lipid parameters (all p > 0.05)<br><br>**Inflammatory parameters:**<br>▪ Statistically significant (p = .039) reduction *versus* baseline in the circulating levels of pro-inflammatory Vascular Endothelial Growth Factor (VEGF) (-10.7%) after SD period.<br>▪ No significant changes to baseline were reported for the remaining pro- and anti-inflammatory cytokines, even if a decreasing trend was observed after both SD and BY (all p values > 0.05).<br>▪ When comparing statistically SD to BY in terms of change to baseline, no significant differences were identified in any of the inflammatory parameters (all p > 0.05)	L
**Bo et al. 2017 (19)**	Italy	RCT DB CO<br>Acute	16 **healthy** subjects<br><br>6 males, 10 females<br><br>Age: 32.3 ± 7.4 y	**Test product(s):** Sourdough bread made with commercial wheat flour, organic wheat flour or organic einkorn flour<br>**Control product(s):** Bread made with commercial wheat flour, no sourdough	**Sourdough:** *L. acidophilus* (strain NM) *L. casei* (strain NM) *S. cerevisiae* (strain NM) *S. exiguous* (strain NM)<br><br>**Control:** NM<br><br>**Authentication**: NM	4-5 h at 28°C and 40% of relative humidity	50 g of starter culture (9.5.10^5 colony forming units/g flour)	**Blood lipids:**<br>▪ FFA AUCs and triglyceride AUCs did not differ by kind of breads (all p > 0.05)	L

Author/ Year/ Reference	Location	Design	Subjects	Interventions	Strains	Fermentation conditions	Doses in starter (CFU/g flour)	Results	Risk of bias
**Darzi et al. 2012 (15)**	United Kingdom	RCT SB CO Acute	20 **healthy** subjects<br><br>9 males, 11 females<br><br>Age: 25.1 ± 4.6 y	White wheat-bread sandwich administered in breakfast:<br>▪ Propionate-rich sourdough bread (2.3 mg per g propionic acid and 2.98 mg per g calcium propionate)<br>▪ Control bread	"Domani starter culture"<br><br>NM<br><br>**Authentication**: NM	NM	NM	▪ Regarding non-esterified fatty acids (NEFA), the area under the curve following SOUR was significantly higher than following control (P = 0.007)<br>▪ The postprandial triglyceride response was not significantly influenced by treatment nor was there a treatment x time effect (data not shown, p value not reported)	**H**
**Korem et al. 2017 (22)**	Israel	RCT<br>1 week	20 **heathy** subjects<br>11 females<br><br>**In test group:**<br>age (y) 39.1 ± 14.3, BMI (kg/m²) 26.5 ± 5.6, Total cholesterol (mg/dL): 191 ± 40<br><br>**In control group:**<br>age (y) 37.3 ± 8.9, BMI (kg/m²) 24.6 ± 5.3, Total cholesterol (mg/dL): 171 ± 26	**Test product:** Whole wheat sourdough bread<br>**Control product:** White wheat bread	NM ("traditional method")<br><br>**Authentication**: NM	NM	NM	**Post hoc analysis:**<br>▪ Significant decrease in levels of the essential minerals calcium, iron, and magnesium and a significant increase in the levels of lactate dehydrogenase (LDH; p < 0.01; Figure 2E), a marker of tissue damage.<br>▪ Significant decrease in the levels of liver enzymes aspartate aminotransferase (AST; p < 0.005; Figure 2B); gamma-glutamyl transpeptidase (GGT; p < 0.005; Figure 2C); alanine aminotransferase (ALT; p < 0.05; Figure 2I), markers of liver function; C-reactive protein (CRP), a commonly used marker of inflammation (p < 0.05; Figure 2K); and creatinine (p < 0.05; Figure 2J), a marker of kidney function.<br>▪ Significant decrease in both total and low-density lipoprotein (LDL) cholesterol levels (p < 0.05 for both; Figure 2 G, H) but no significant change in high-density lipoprotein (HDL) cholesterol levels (Figure 2L).	**L**
**Maioli et al. 2008 (23)**	Italy	RCT CO Acute	16 subjects with **impaired glucose tolerance (IGT)**<br><br>9 males, 7 females	▪ **Test product:** Wheat / cornflour (70:30) sourdough bread<br>▪ **Control product(s):** Wheat / cornflour (70:30) leavened with yeast	*S. cerevisiae, L. brevis SB3, L. plantarum SB24*, isolated from natural sourdough	Proofed for 8 h, 30°C, 90% humidity	NM	**Other markers:**<br>▪ We did not find any significant difference in plasma cholesterol and triglycerides after consuming the two types of meals (p values not mentioned).	**M**

Author/ Year/ Reference	Location	Design	Subjects	Interventions	Strains	Fermentation conditions	Doses in starter (CFU/g flour)	Results	Risk of bias
			Age: 52-75 y		**Authentication**: PCR test reported in a cited study				
**Tucker et al. 2014 (26)**	Canada	RCT CO Acute	12 **type-2 diabetes (T2D)** subjects<br><br>11 males, 1 female<br><br>Age: 63.8 ± 3.30 y<br>BMI: 32.5 ± 1.41 kg/m²<br>HbA1c: 6.94 ± 0.41%<br>Fasting blood glucose: 6.88 ± 0.58 mmol/L	**Treatment breads:**<br>▪ sprouted sourdough (wheat; SPR),<br>▪ whole-grain sourdough (mainly wheat; WG),<br>▪ enriched white sourdough (wheat flour enriched with added lactic acid, ascorbic acid and sodium acetate; SD),<br>▪ enriched white (same as SD without sourdough; WB).	Not known (Commercial breads)<br><br>**Authentication**: NM	Not known (Commercial breads)	Not known (Commercial breads)	**Serum lipids:**<br>▪ Incremental AUC data for the time period following the bread challenge (0–180 min) showed that LDL-cholesterol was significantly lower ($p \leq 0.05$) with consumption of sourdough white bread compared to whole grain sourdough, but there were no significant changes in other serum lipids (all p> 0.05).<br>▪ Incremental AUC data for the time period following the second meal challenge (180–300 min) showed that apolipoprotein B-100 was significantly higher ($p \leq 0.05$) with previous consumption of white sourdough compared to white bread, but there were no significant changes in other serum lipids (all p> 0.05).<br>▪ Incremental AUC data for the entire postprandial period (0–300 min) showed no significant differences in any serum lipid endpoints between the four treatment breads (all p> 0.05).	L

Abbreviations AUC: Area Under the Curve; BMI: Body Mass Index; CFU: Colony Forming Unit; CHO: Carbohydrates; CO: Crossover; CS: Celiac sprue; DB: Double-Blind; GCV: Gastric Content Volume; GI: Gastrointestinal Index; HbA1c: glycated hemoglobin; iAUC: incremental Area Under the Curve; IBS-SS: Irritable Bowel Severity Scoring System; NM: not mentioned; RCT: Randomized Controlled Trial; SB: Single-Blind; TGV: Total Gastric Volume; VAS: Visual analogue Scale.
