# Supplementary material for: Nutritional benefits of sourdoughs: A systematic review
Source: Adv Nutr. 2022 Dec 16;14(1):22–9. doi: 10.1016/j.advnut.2022.10.003 (PMC10103004; doi:10.1016/j.advnut.2022.10.003)
Supplement: Multimedia component 2 [file mmc2.pdf]

Supplementary methods

Bibliography used for the systematic review

11. Liljeberg HGM, Lönner CH, Björck IME. Sourdough fermentation or addition of organic acids or corresponding salts to bread improves nutritional properties of starch in healthy humans. JOURNAL OF NUTRITION 1995; 125.
12. Scazzina F, Rio Dd, Pellegrini N, Brighenti F. Sourdough bread: starch digestibility and postprandial glycemic response. JOURNAL OF CEREAL SCIENCE 2009; 49.
13. Rizzello CG, Portincasa P, Montemurro M, Di Palo DM, Lorusso MP, Angelis M de, Bonfrate L, Genot B, Gobbetti M. Sourdough Fermented Breads are More Digestible than Those Started with Baker's Yeast Alone: An In Vivo Challenge Dissecting Distinct Gastrointestinal Responses. Nutrients 2019; 11.
14. Dall'Asta M, Angelino D, Paolella G, Dodi R, Pellegrini N, Martini D. Nutritional Quality of Wholegrain Cereal-Based Products Sold on the Italian Market: Data from the FLIP Study. Nutrients 2022; 14.
15. Darzi J, Frost GS, Robertson MD. Effects of a novel propionate-rich sourdough bread on appetite and food intake. Eur J Clin Nutr 2012; 66:789–94.
16. Juntunen KS, Laaksonen DE, Autio K, Niskanen LK, Holst JJ, Savolainen KE, Liukkonen K-H, Poutanen KS, Mykkänen HM. Structural differences between rye and wheat breads but not total fiber content may explain the lower postprandial insulin response to rye bread. Am J Clin Nutr 2003; 78:957–64.
17. Novotni D, Cukelj N, Smerdel B, Bituh M, Dujmic F, Curic D. Glycemic index and firming kinetics of partially baked frozen gluten-free bread with sourdough. JOURNAL OF CEREAL SCIENCE 2012; 55.
18. Zamaratskaia G, Johansson DP, Junqueira MA, Deissler L, Langton M, Hellström PM, Landberg R. Impact of sourdough fermentation on appetite and postprandial metabolic responses - a randomised cross-over trial with whole grain rye crispbread. The British journal of nutrition 2017; 118.
19. Bo S, Seletto M, Choc A, Ponzo V, Lezo A, Demagistris A, Evangelista A, Ciccone G, Bertolino M, Cassader M, et al. The acute impact of the intake of four types of bread on satiety and blood concentrations of glucose, insulin, free fatty acids, triglyceride and acylated ghrelin. A randomized controlled cross-over trial. FOOD RESEARCH INTERNATIONAL 2017; 92.
20. Bondia-Pons I, Nordlund E, Mattila I, Katina K, Aura AM, Kolehmainen M, Oresic M, Mykkanen H, Poutanen K. Postprandial differences in the plasma metabolome of healthy Finnish subjects after intake of a sourdough fermented endosperm rye bread versus white wheat bread. NUTRITION JOURNAL 2011; 10.
21. Lappi J, Mykkänen H, Knudsen KEB, Kirjavainen P, Katina K, Pihlajamäki J, Poutanen K, Kolehmainen M. Postprandial glucose metabolism and SCFA after consuming wholegrain rye bread and wheat bread enriched with bioprocessed rye bran in individuals with mild gastrointestinal symptoms. NUTRITION JOURNAL 2014; 13.
22. Korem T, Zeevi D, Zmora N, Weissbrod O, Bar N, Lotan-Pompan M, Avnit-Sagi T, Kosower N, Malka G, Rein M, et al. Bread Affects Clinical Parameters and Induces Gut Microbiome-Associated Personal Glycemic Responses. Cell metabolism 2017; 25.
23. Maioli M, Pes GM, Sanna M, Cherchi S, Dettori M, Manca E, Farris GA. Sourdough-leavened bread improves postprandial glucose and insulin plasma levels in subjects with impaired glucose tolerance. ACTA DIABETOLOGICA 2008; 45.

24. MacKay KA, Tucker AJ, Duncan AM, Graham TE, Robinson LE. Whole grain wheat sourdough bread does not affect plasminogen activator inhibitor-1 in adults with normal or impaired carbohydrate metabolism. Nutrition, Metabolism and Cardiovascular Diseases 2012; 22:704–11.
25. Tucker AJ, MacKay KA, Robinson LE, Graham TE, Bakovic M, Duncan AM. The effect of whole grain wheat sourdough bread consumption on serum lipids in healthy normoglycemic/normoinsulinemic and hyperglycemic/hyperinsulinemic adults depends on presence of the APOE E3/E3 genotype: a randomized controlled trial. Nutrition & Metabolism 2010; 7.
26. Tucker AJ, Vandermey JS, Robinson LE, Graham TE, Bakovic M, Duncan AM. Effects of breads of varying carbohydrate quality on postprandial glycaemic, incretin and lipidaemic response after first and second meals in adults with diet-controlled type 2 diabetes. JOURNAL OF FUNCTIONAL FOODS 2014; 6.
27. Najjar AM, Parsons PM, Duncan AM, Robinson LE, Yada RY, Graham TE. The acute impact of ingestion of breads of varying composition on blood glucose, insulin and incretins following first and second meals. The British journal of nutrition 2009; 101:391–8.
28. Mofidi A, Ferraro ZM, Stewart KA, Tulk HMF, Robinson LE, Duncan AM, Graham TE. The acute impact of ingestion of sourdough and whole-grain breads on blood glucose, insulin, and incretins in overweight and obese men. Journal of Nutrition and Metabolism 2012; 2012.
29. Iversen KN, Johansson D, Brunius C, Andlid T, Andersson R, Langton M, Landberg R. Appetite and Subsequent Food Intake Were Unaffected by the Amount of Sourdough and Rye in Soft Bread-A Randomized Cross-Over Breakfast Study. Nutrients 2018; 10.
30. Polese B, Nicolai E, Genovese D, Verlezza V, La Sala CN, Aiello M, Inglese M, Incoronato M, Sarnelli G, Rosa T de, et al. Postprandial Gastrointestinal Function Differs after Acute Administration of Sourdough Compared with Brewer's Yeast Bakery Products in Healthy Adults. J Nutr 2018; 148:202–8.
31. Laatikainen R, Koskenpato J, Hongisto S-M, Loponen J, Poussa T, Hillilä M, Korpela R. Randomised clinical trial: low-FODMAP rye bread vs. regular rye bread to relieve the symptoms of irritable bowel syndrome. Aliment Pharmacol Ther 2016; 44:460–70.
32. Laatikainen R, Koskenpato J, Hongisto SM, Loponen J, Poussa T, Huang X, Sontag-Strohm T, Salmenkari H, Korpela R. Pilot study: comparison of sourdough wheat bread and yeast-fermented wheat bread in individuals with wheat sensitivity and irritable bowel syndrome. Nutrients 2017; 9.
33. Calasso M, Francavilla R, Cristofori F, Angelis M de, Gobbetti M. New Protocol for Production of Reduced-Gluten Wheat Bread and Pasta and Clinical Effect in Patients with Irritable Bowel Syndrome: A randomised, Double-Blind, Cross-Over Study. Nutrients 2018; 10.
34. Pagliai G, Venturi M, Dinu M, Galli V, Colombini B, Giangrandi I, Maggini N, Sofi F, Granchi L. Effect of consumption of ancient grain bread leavened with sourdough or with baker's yeast on cardio-metabolic risk parameters: a dietary intervention trial. INTERNATIONAL JOURNAL OF FOOD SCIENCES AND NUTRITION 2020.
49. Fredensborg MH, Perry T, Mann JIM, Chisholm A, Rose M. Rising methods and leavening agents used in the production of bread do not impact the glycaemic index. Asia Pacific Journal of Clinical Nutrition 2010; 19.
