# Supplementary material for: Nutritional benefits of sourdoughs: A systematic review
Source: Adv Nutr. 2022 Dec 16;14(1):22–9. doi: 10.1016/j.advnut.2022.10.003 (PMC10103004; doi:10.1016/j.advnut.2022.10.003)

Supplementary Figure 1: Quality assessment tool …………………………………………………………………………………… p. 2

Supplementary Figure 2: Quality assessment of all 25 eligible studies included in the qualitative Analyses …………. p. 3

Supplementary Figure 3: Detailed flow chart with exclusions ……………………………………………………….……. p. 4

Supplementary Figure 1

Quality assessment tool

The tool used estimated the potential bias arising for each of the individual component indicated below.

**Quality criteria**	**Risk of bias**		
	**Low (1 point)**	**Medium (0.5 point)**	**High (0 point)**
Randomization & allocation concealment	Adequately reported & performed	Partially done or reported	No randomization
Blinding of participants and assessors	Blinding of both participants and personnel with adequate method	Partial blinding or method not reported	No blinding
Choice of comparator	Comparator is adequate to bring evidence that the effect is induced by the intervention component alone	The comparator varies from the test product in other aspects than the intervention component	No comparator
Length of assessment period	The length is sufficient to observe a clinical effect on the primary outcome	Concerns regarding study assessment period	Insufficient study period for demonstrating an effect on the outcome
Study groups details & comparability	Information on baseline characteristics are available per group, and no significant differences were reported	Information lacking, or participants differ in characteristics, but unrelated to the primary outcome	Study groups differ in their baseline characteristics
Handling of missing outcomes data	No missing outcomes, or the missing outcomes are reported and accounted for	Lack of information related to the handling of missing outcomes data	Uneven loss to follow-up between groups and no strategy for handling
Measurement methods	The marker is recognized as gold standard and the measurement method is adequate	The marker is not recognized as gold standard, or is a surrogate	Inappropriate marker / method for measuring the outcome

The risk of bias was calculated as the total score divided by the number of parameters (n=7), and the following classification was used:

**Final score**	**Risk of bias**
< 40%	High
40-70%	Medium
> 70%	Low

Supplementary Figure 2

Quality assessment of all 25 eligible studies included in the qualitative analyses

Authors, Year, Reference	Randomization & allocation concealment	Blinding of participants and assessors	Choice of comparator	Study duration	Baseline characteristics	Missing outcomes data	Measurement methods	Overall risk of bias
Bo *et al.* 2017 (19)	green	green	green	green	green	green	green	L
Bondia-Pons *et al* 2011 (20)	red	red	red	red	red	green	green	H
Dall'Asta *et al.* 2022 (14)	yellow	red	green	green	red	green	green	M
Darzi *et al.* 2012 (15)	yellow	red	red	yellow	yellow	yellow	yellow	H
Fredensborg *et al.* 2010 (49)	yellow	red	green	green	green	yellow	green	L
Korem *et al.* 2017 (22)	green	red	green	green	green	green	green	L
Lappi *et al.* 2014 (21)	green	red	green	green	yellow	green	yellow	L
Liljeberg *et al.* 1995 (11)	yellow	red	yellow	green	red	red	green	M
Maioli *et al.* 2008 (23)	yellow	red	green	green	red	yellow	green	M
Najjar *et al.* 2009 (27)	yellow	yellow	green	green	yellow	green	green	L
Mofidi *et al.* 2012 (28)	yellow	yellow	green	green	yellow	green	green	L
Polese *et al.* 2018 (30)	green	green	green	green	red	green	green	L
Tucker *et al.* 2010 (25)	yellow	red	red	green	red	green	green	M
Tucker *et al.* 2014 (26)	yellow	red	green	green	green	yellow	green	L
Zamaratskaia *et al.* 2017 (18)	green	yellow	green	green	green	green	green	L
Rizzello *et al.* 2019 (13)	yellow	green	green	green	yellow	yellow	green	L
Iversen *et al.* 2018 (29)	green	red	green	green	green	green	green	L
MacKay *et al.* 2012 (24)	green	red	green	green	green	green	green	L
Scazzina *et al.* 2009 (12)	yellow	red	green	green	red	red	red	H
Laatikainen *et al.* 2016 (31)	green	green	green	green	green	red	green	L
Laatikainen *et al.* 2017 (32)	green	green	green	green	yellow	green	yellow	L
Pagliai *et al.* 2020 (34)	yellow	green	green	green	yellow	green	yellow	L
Calasso *et al.* 2018 (33)	green	green	green	green	yellow	yellow	green	L
Novotni *et al.* 2012 (17)	red	red	green	green	red	red	red	H
Juntunen *et al.* 2003 (16)	red	red	yellow	green	yellow	yellow	green	M

green	Low risk of bias (1 point)
yellow	Medium risk of bias (0.5 point)
red	High risk of bias (0 point)

Supplementary Figure 3

Detailed flow chart with exclusions

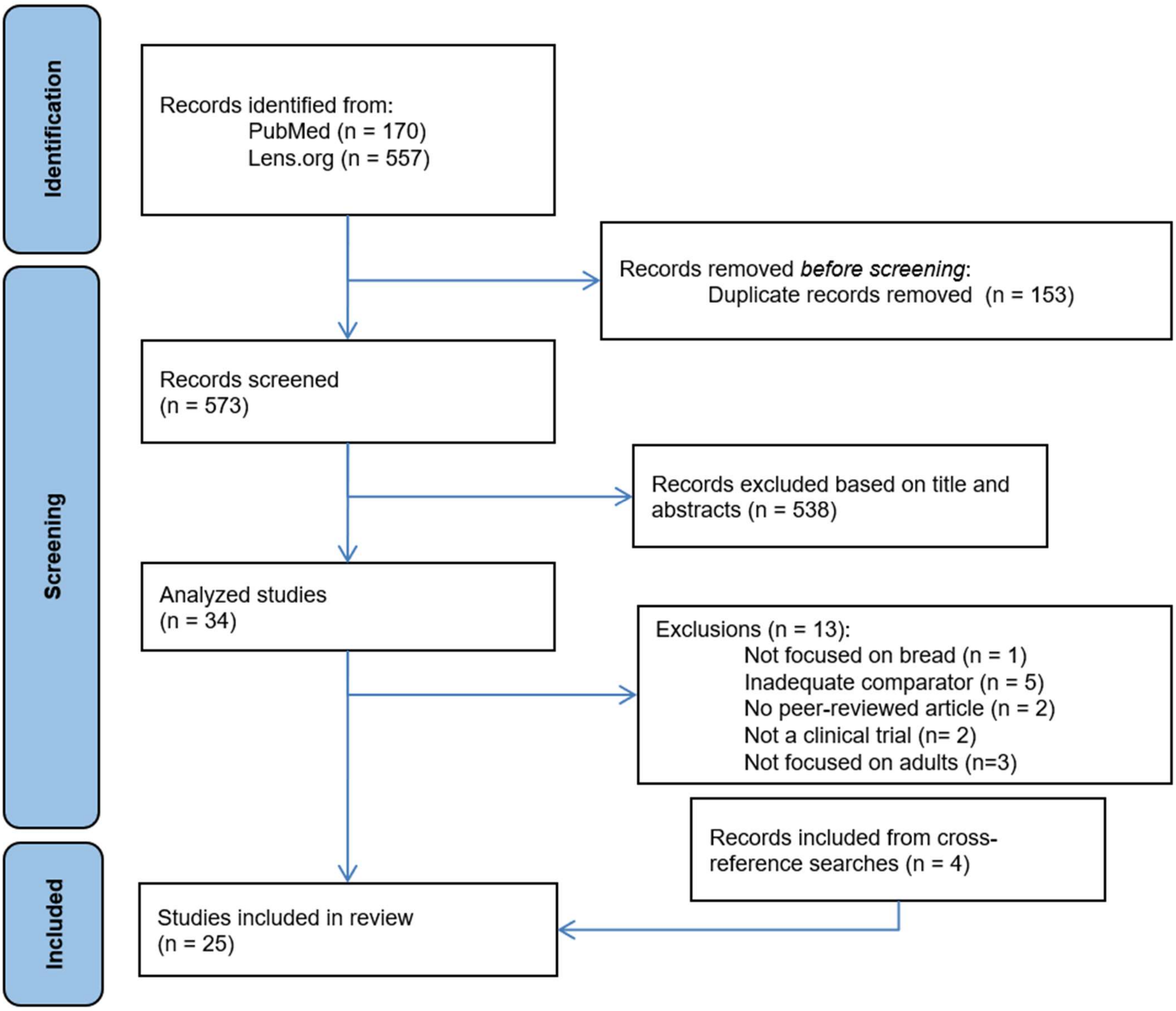

Supplement: Multimedia component 3 [file mmc3.pdf]
